# A role of pigment epithelium-derived factor in zinc-mediated mechanism of neurodegeneration in glaucoma

**DOI:** 10.1038/s42003-025-08370-8

**Published:** 2025-07-01

**Authors:** Dmitry V. Chistyakov, Anatolii S. Belousov, Marina P. Shevelyova, Elena N. Iomdina, Viktoriia E. Baksheeva, Natalia G. Shebardina, Anastasia M. Moysenovich, Timofey K. Bulgakov, Sergey Yu. Petrov, Mikhail L. Shishkin, Sanchay S. Tulush, Veronika V. Tiulina, Ekaterina I. Pogodina, Olga S. Gancharova, Olga M. Filippova, Alexey V. Baldin, Sergey V. Goriainov, Arina I. Nikolskaya, Arthur O. Zalevsky, Andrei A. Deviatkin, Alisa A. Vologzhannikova, Neonila V. Gorokhovets, Ekaterina A. Litus, Sergey V. Komarov, François Devred, Marina G. Sergeeva, Alexey V. Mishin, Sergey S. Bukhdruker, Lijie Wu, Evandro A. Araujo, Andrey A. Zamyatnin, Ivan I. Senin, Dmitry V. Zinchenko, Philipp O. Tsvetkov, Valentin I. Borshchevskiy, Sergei E. Permyakov, Evgeni Yu. Zernii

**Affiliations:** 1 Belozersky Institute of Physico-Chemical Biology, Lomonosov Moscow State University, Moscow, Russia; 2 Peoples’ Friendship University of Russia (RUDN University), Moscow, Russia; 3 Moscow Institute of Physics and Technology (National Research University), Dolgoprudniy, Russia; 4 Sao Carlos Institute of Physics, University of Sao Paulo, poloTErRA, Sao Carlos São Paolo, Brazil; 5 Institute for Biological Instrumentation, Pushchino Scientific Center for Biological Research of the Russian Academy of Sciences, Pushchino, Russia; 6 Helmholtz National Medical Research Center of Eye Diseases, Moscow, Russia; 7 Institut Neurophysiopathol, INP, Faculté des Sciences Médicales et Paramédicales, Aix Marseille Univ, CNRS, Marseille, France; 8 Faculty of Biology, Lomonosov Moscow State University, Moscow, Russia; 9 Department of Biological Chemistry, Sechenov First Moscow State Medical University, Moscow, Russia; 10 Branch of Shemyakin and Ovchinnikov Institute of Bioorganic Chemistry, Russian Academy of Sciences, Pushchino, Russia; 11 Faculty of Bioengineering and Bioinformatics, Lomonosov Moscow State University, Moscow, Russia; 12 Shemyakin-Ovchinnikov Institute of Bioorganic Chemistry of the Russian Academy of Sciences, Moscow, Russia; 13 Institute of Translational Medicine and Biotechnology, Sechenov First Moscow State Medical University, Moscow, Russia; 14 Skryabin Moscow State Academy of Veterinary Medicine and Biotechnology, Moscow, Russia; 15 iHuman Institute, ShanghaiTech University, Shanghai, China; 16 Brazilian Synchrotron Light Laboratory (LNLS), Brazilian Center for Research in Energy and Materials, Campinas, São Paulo, Brazil; 17 Joint Institute for Nuclear Research, Dubna, Russia

**Keywords:** Retina, Neurotrophic factors, Metalloproteins, X-ray crystallography, Glaucoma

## Abstract

Glaucoma is a neurodegenerative condition involving optic nerve damage and retinal ganglion cells death. Animal studies suggested that the pathway linking these events can be mediated by mobile zinc secreted into the intraretinal space and exerting cytotoxic effects. Whether this mechanism is relevant for human glaucoma and what are the targets of extracellular zinc is unknown. We report that increased zinc content in the aqueous humor and retina is indeed a characteristic of glaucomatous neuropathy, and excess extracellular zinc may be recognized by the key retinal neurotrophic factor PEDF. Biophysical and X-ray crystallographic studies show that PEDF coordinates zinc ions in five types of intermolecular high-affinity sites, leading to a decrease in negative surface charge and reversible oligomerization of the protein, thereby masking the target recognition sites responsible for its neurotrophic and antiangiogenic activities and collagen binding. Notably, PEDF secretion is enhanced in both glaucoma and retinal cell models in response to zinc stress; however, zinc binding negatively affects axogenic, differentiative and prosurvival functions of PEDF by suppressing its ability to activate receptor PEDF-R/PNPLA2. We suggest that glaucomatous neurodegeneration is associated with direct inhibition of PEDF signaling by extracellular zinc, making their complex a promising target for neuroprotective therapy.

## Abstract (web summary)

Extracellular zinc release in glaucoma may affect the key retinal neurotrophic factor PEDF, which binds zinc at intermolecular sites and forms dimers and trimers, losing the ability to recognize PEDF-R and exhibit neurotrophic and antiangiogenic activity.

## Introduction

Zinc is a key micronutrient that is essential for many aspects of neuronal function, such as direct mediation of neurotransmission^1,2^. Although the total concentration of zinc in the nervous system is remarkably high, healthy neurons contain only trace amounts of free Zn^2+^ since its excess is excreted from the cytoplasm by specific Zn^2+^-transporting proteins (ZnTs) into mitochondria, lysosomes or specific vesicles (zincosomes), and/or coordinated by Zn^2+^-buffering proteins (e.g., metallothioneins)^2^. The remaining zinc is incorporated into complexes with numerous Zn^2+^-binding proteins and, depending on binding affinity, can play a structural and functional role or exhibit signaling activity by being redistributed among signal transduction proteins (so-called “mobile” or “loosely bound” zinc)^3–5^. When zinc homeostasis is disturbed, it becomes a pathological factor causing irreversible neurodegenerative changes in the central nervous system and retina^4,6^. In particular, the pronounced aberrant activity of excess zinc may be realized in the interneuron space. Even under normal conditions, zinc concentration in the synaptic cleft can transiently increase from 15–20 nM to 10–300 μM and disruption of its reuptake can significantly affect cell viability^2,5,7^.

Glaucoma is a neurodegenerative disease, one of the leading causes of irreversible blindness. It has a complex pathogenesis, which is always characterized by damage to the optic nerve with subsequent death of retinal ganglion cells (RGCs), the projection neurons of the eye. The most common form of the disease is primary open-angle glaucoma (POAG), the main risk factor for which is a transient or persistent increase in intraocular pressure (IOP) caused by obstruction of aqueous humor (AH) outflow through the trabecular meshwork and Schlemm’s canal in the anterior chamber angle and/or through the uveoscleral pathway. Chronic ocular hypertension leads to cupping of the optic nerve head into the lamina cribrosa and subsequent death of RGCs due to the inability to regenerate their axons constituting the optic nerve^8^. Early stages of POAG are asymptomatic and the emerging disease can be recognized only using sophisticated biomarker-based approaches^9,10^. Meanwhile, manifestation of the symptoms, such as narrowing of the visual fields, indicates an irreversible neurodegenerative process, which is poorly treated. It is noteworthy that the multifactorial nature of POAG hinders the development of targeted regenerative therapy, which it currently limited to the improvement of AH outflow and IOP reduction^9^. Therefore, it is important to characterize the entire chain of the molecular events linking ocular hypertension and RGCs death in order to find the most sensitive and convenient target(s) for neuroprotective therapeutic intervention.

According to studies in animal models of glaucoma, the progressive RGCs death occurs mainly via apoptosis triggered by several interrelated mechanisms, including mechanical stress and impaired axonal transport of neurotrophic factors, glutamate excitotoxicity, as well as retinal ischemia and oxidative stress^8^. Recently, using a rat model of optic nerve crush, it has been demonstrated that the primary factor mediating RGSs degeneration under these conditions is mobile zinc. Shortly after the optic nerve injury, mobile zinc accumulates in retinal amacrine cells and is secreted towards RGCs, inducing their degeneration, while its capture by zinc-specific chelators attenuates RGCs apoptosis. The increase in zinc concentration and RGCs death can be suppressed by both membrane-permeable and membrane-impermeable zinc chelators, indicating that mobile cytotoxic zinc functions both in the cell and in the interneuronal space^11^. The mechanism underlying secretion of cytotoxic zinc by amacrine cells has been characterized^12^. However, whether this mechanism is relevant for human glaucoma and what are the targets of extracellular zinc remains unknown.

The goal of this study was to characterize zinc homeostasis in the glaucomatous eye, including the identification of molecules governing its bioavailability, and to reveal protein targets of extracellular zinc, focusing on signaling proteins that may mediate its neurotoxic effects in the retina. Particularly, we employed atomic absorption spectroscopy, gas chromatography–mass spectrometry and structural bioinformatics approaches to determine changes in the content of zinc and Zn^2+^-binding molecules (low-molecular-weight compounds and proteins) secreted into AH in patients with different stages of POAG and animals with an ocular hypertension model. Indeed, due to the vitreal-aqueous gradient, changes in the retinal secretome may reflect alterations in composition of AH, which is increasingly being used for proteomic and metabolomic analysis of retinal diseases, including glaucoma^9,13–15^. We next examined the Zn^2+^-dependent proteome of AH and screened it for the proteins capable of mediating cytotoxic signals of zinc, among which pigment epithelium-derived factor (PEDF) was the most relevant candidate. The zinc binding affinity and Zn^2+^-dependent structural properties of PEDF were characterized in vitro by biophysical and X-ray crystallographic studies with ~2 Å-resolution. Finally, secretion and neurotrophic activity of PEDF under zinc stress conditions were investigated in retinal cells models using various approaches including flow cytometry, confocal microscopy, and targeted liquid chromatography–mass spectrometry. Our results suggest that a crucial neurodegeneration pathway in glaucoma may involve the effect of extracellular zinc on the activity of the neurotropic factor PEDF and consequently its receptor PEDF-R, which may thus represent relevant targets for neuroprotective therapy.

## Results

### Glaucoma development in patients is associated with accumulation of zinc in AH

**Fig. 1:**
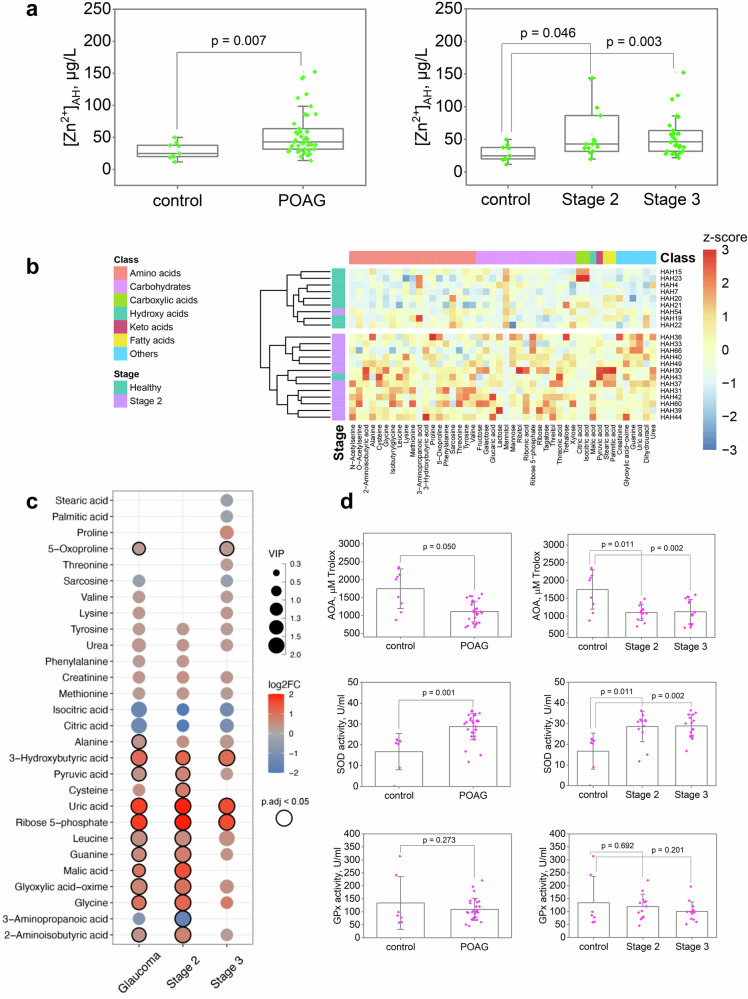
Glaucoma is characterized by altered zinc homeostasis and oxidative stress in patients. **a** Total zinc content in AH of participants (Supplementary Data 1) was analyzed using AAS. Zinc concentration significantly increased from as early as stage 2 of POAG (*p* < 0.05). **b,** **c** Identifi**c**ation of low molecular weight zinc chelators in AH by full-scale metabolomic (GC-MS) analysis. **b** Heat map illustrating changes in the concentration of AH metabolites in stage 2 POAG. Mean-centered and unit variance scaled concentrations (z-scores) for 46 metabolites with *p* < 0.05 and/or VIP score > 1 are shown. **c** Zinc-binding metabolites in AH showing alterations in glaucoma. The dot plot illustrates the binary logarithm of fold change (FC), the significance of the variable in prediction (VIP), and the false discovery rate (FDR)-adjusted *p*-values (p.adj) for the major zinc chelators relative to the control group. The points with *p* < 0.05 and/or VIP score > 1 are highlighted. The general trend among the most affinity zinc chelators is an increase in amino acids and a decreased in citrate and isocitrate. **d** Markers of oxidative stress in AH were evaluated by colorimetric methods. Glaucoma is associated with oxidative stress manifested in AH as a significant decrease in AOA with a compensatory increase in Zn^2+^-dependent SOD (*p* < 0.05), but without a significant change in GPx activity. The data in **a** and **d** are presented as mean and error bars indicate SEM.

Zinc content was examined in AH of a cohort of patients with different stages of POAG or cataract (control; Supplementary Data 1, 2) using atomic absorption spectrometry (AAS). The mean zinc concentration in the control group was 29 ± 4 μg/L (0.44 μM). In patients with POAG stage 2 and 3, it increased to 58 ± 11 μg/L (0.89 μM) and 56 ± 6 μg/L (0.86 μM), respectively, i.e., these values were significantly different from control but not different from each other (Fig. 1a). For comparison with previous data, we also calculated the zinc content in total POAG regardless its stage, which was 55 ± 5 μg/L (0.84 μM), about 2-fold higher than in control. Notably, in AH of some patients, zinc concentration reached 2.0-2.3 µM (5 cases) or even 7 µM (1 case). No effect of any of the antiglaucomatous treatments on the content of Zn^2+^ in AH was detected (Supplementary Data 3). Thus, it was established that zinc concentration in AH increases following the progression of the pathological process in POAG.

### Glaucoma development in patients is associated with alterations in zinc chelators in AH

AH consists of a broad range of low-molecular-weight compounds, among which may be zinc chelators^15^. To assess possible POAG-associated changes in these chelators, we performed metabolomic analysis of AH of patients in our control and POAG groups focusing on molecules able to bind zinc. A total of 81 metabolites were identified (Supplementary Data 4). Statistical analysis revealed 46 metabolites characterized by significant changes in POAG, which included 19 amino acids, 4 other organic acids (citric, isocitric, malic and pyruvic acids), 15 carbohydrates, 4 organoheterocyclic compounds, 2 fatty acids (palmitic and stearic acids), 1 organosilicon compound (glyoxylic acid-oxime) and urea (Supplementary Data 5–8, Supplementary Fig. 1, Table 1). Among them, 22 compounds demonstrated alterations at all stages of glaucoma, 8 compounds differed from the control group only in early POAG (stage 2), whereas other 16 compounds were altered only in advanced POAG (stage 3). The changes in the detected compounds were independent of the antiglaucoma therapy used (Supplementary Data 9). In general, AH concentration of amino acids, carbohydrates, and heterocycles increased, whereas the content of carboxylic acids decreased in glaucoma, especially in stage 2 of the disease (Fig. 1b). The additional compounds showing decreased levels in glaucoma included 3-aminopropanoic acid, sarcosine, mannitol, and fatty acids. The most pronounced effects were found in the case of ribose-5-phosphate, threitol, and uric acid (4-fold increase), as well as citrate (4-fold decrease).

**Table 1:** AH metabolites/zinc chelators associated with POAG identified by GC-MS-based metabolomic analysis

#	Metabolite	Class	Fold change in POAG versus control according to this study ^*a*^			Zinc binding ^*b*^ and AH concentration (C_AH_) according to databases ^*c*^ or (reference)			
			stage 2	stage 3	total	+/n.d. ^*d*^	K_D_^1^, M	K_D_^2^, M	C_AH_, mM
1	N-Acetylserine	Amino acids	3.54	1.76	2.36	n.d.	-	-	-
2	O-Acetylserine		-	1.22	1.20	n.d.	-	-	-
3	2-Aminoisobutyric acid		1.63	1.19	1.34	+ ^*c*^	2.82E−05	2.51E−09	-
4	Alanine		1.38	1.27	1.30	+ ^*c*^	2.34E−05	2.19E−09	0.34^137^
5	Cysteine		1.70	-	1.37	+ ^*c*^	7.76E−10	7.59E−19	0.01^137^
6	Glycine		2.24	1.79	1.94	+ ^*c*^	9.33E−06	5.89E−10	0.02^137^
7	Isobutyrylglycine		-	1.22	1.20	n.d.	-	-	-
8	Leucine		1.42	1.39	1.40	+ ^*c*^	2.75E−05	1.82E−09	0.14^137^
9	Lysine		-	1.26	1.27	+ ^*c*^	8.71E−05	1.38E−08	0.15^137^
10	Methionine		1.26	1.18	1.21	+ ^*c*^	4.17E−05	4.47E−09	0.04^137^
11	3-Aminopropanoic acid		0.33	-	0.56	+ ^*c*^	7.08E−05	-	-
12	3-Hydroxybutyric acid		2.27	2.05	2.13	+ ^*c*^	0.100	0.019498	0.03
13	Proline		-	1.51	1.40	+ ^*c*^	7.41E−06	2.04E−10	0.02^137^
14	5-Oxoproline		-	1.25	1.21	+^138^	-	-	-
15	Phenylalanine		1.27	-	1.19	+ ^*c*^	6.03E−05	6.03E−09	0.11^137^
16	Sarcosine		-	0.73	0.76	+ ^*c*^	2.95E−05	5.01E−09	-
17	Threonine		-	1.22	1.20	+ ^*c*^	2.88E−05	4.47E−09	0.15^137^
18	Tyrosine		1.24	1.20	1.22	+ ^*c*^	6.17E−05	3.09E−09	0.11^137^
19	Valine		-	1.19	1.19	+ ^*c*^	3.39E−05	5.75E−09	0.34^137^
20	Citric acid	Carboxylic acids	0.26	0.47	0.40	+ ^*c*^	1.91E−05	4.37E−08	0.18^139^
21	Isocitric acid		0.25	0.42	0.37	+^140^	7.04E−06	-	0.01^139^
22	Malic acid	Hydroxy acids	2.83	-	1.87	+ ^*c*^	0.001	5.01E−06	0.01^139^
23	Pyruvic acid	Keto acids	1.56	1.22	1.33	+ ^*c*^	0.069	0.011	0.14^139^
24	Fructose	Carbohydrates	-	1.23	1.25	n.d.	-	-	0.03^141^
25	Galactose		1.35	-	1.33	n.d.	-	-	-
26	Glucaric acid		-	1.42	1.42	+^142^	3.47E−4	-	-
27	Lactose		2.00	0.51	2.04	n.d.	-	-	-
28	Mannitol		0.14	0.51	0.39	n.d.	-	-	-
29	Mannose		1.45	1.32	1.36	n.d.	-	-	-
30	Ribitol		-	1.27	1.25	n.d.	-	-	-
31	Ribonic acid		-	1.44	1.38	n.d.	-	-	-
32	Ribose 5-phosphate		4.04	2.92	3.29	+ ^*c*^	6.31E−3	-	-
33	Ribose		-	1.20	1.17	n.d.	-	-	-
34	Tagatose		1.42	1.27	1.32	n.d.	-	-	-
35	Threitol		3.95	1.89	2.58	n.d.	-	-	-
36	Threonic acid		1.69	-	1.36	n.d.	-	-	-
37	Trehalose		-	1.63	1.57	n.d.	-	-	-
38	Xylose		1.27	1.25	1.26	n.d.	-	-	-
39	Guanine	Purines	1.72	1.36	1.48	+ ^*c*^	0.617	-	-
40	Uric acid		3.92	2.87	3.22	+^143^	-	-	0.10^55^
41	Dihydrouracil	Pyrimidines	1.33	-	1.33	n.d.	-	-	-
42	Creatinine	Imidazoles	1.38	1.27	1.31	+^144^	-	-	0.03^145^
43	Stearic acid	Fatty acids	-	0.78	0.86	+^146^	-	-	-
44	Palmitic acid		-	0.82	0.90	+^146^	-	-	-
45	Glyoxylic acid-oxime	Organosilicons	1.95	1.39	1.58	+^147^	-	-	-
46	Urea	Ureas	1.32	1.24	1.27	+ ^*c*^	-	-	4.40^137^

^*a*^Metabolites characterized by significant (*p* < 0.05 and/or VIP score > 1) changes in POAG (for details, see Supplementary Data 4-9).;

^*b*^Data for ZnL (K_D_^1^) and ZnL_2_ (K_D_^2^) complexes.;

^*c*^According to SC database and/or NIST46 database (version 8.0).;

^d^No data.

Analysis of the metabolites exhibiting significant alterations in POAG showed that zinc chelators among them are all amino acids, other organic acids, fatty acids, as well as heterocyclic compounds, namely guanine and uric acid (Fig. 1c, Table 1). Amino acids (2-aminoisobutyric acid, alanine, cysteine, glycine, leucine, lysine, methionine, proline, phenylalanine, sarcosine, threonine, tyrosine, and valine) and carboxylic acids (citric acid and isocitric acid) can form complexes ZnL and/or ZnL_2_. Most of these amino acids exhibited an increase, while citric and isocitric acids showed a pronounce decrease in POAG, the most noticeably in stage 2. We speculated that the increased extracellular zinc in POAG may redistribute from citrate/isocitrate to amino acid complexes, especially in the early phases of the disease. It should be added that anti-glaucomatous therapy did not affect the content of zinc chelators in AH (Supplementary Data 9).

### Glaucoma development in patients is associated with an increase in oxidative stress markers in AH

A common cause of zinc release is the oxidation of thiol groups of Zn^2+^-coordinating cysteines of Zn^2+^-buffer proteins under conditions of oxidative stress^16^. To find out whether oxidative stress accompanies the increase in zinc concentration that was detected in POAG patients, we used randomly selected AH samples to analyze total antioxidant activity (AOA; the more severe oxidative stress, the less residual AOA), mediated by low-molecular-weight antioxidants, and the activity of key antioxidant defense enzymes, superoxide dismutase (SOD) and glutathione peroxidase (GPx) (Fig. 1d). AOA was significantly lowered in POAG patients as early as at stage 2 and remained low at stage 3 of the disease, indicating a pronounced oxidative stress. At the same time, we found a compensatory increase in SOD activity in AH in the absence of significant change in GPx activity. These observations are also true for specific activity of these enzymes, as we did not observe significant changes in total protein in AH associated with POAG. Thus, the oxidative stress manifesting in AH is one of the characteristic processes in POAG, which may be responsible for the observed increase in AH zinc concentration.

### Zinc release and oxidative stresses accompany glaucoma neuropathy caused by ocular hypertension

**Fig. 2:**
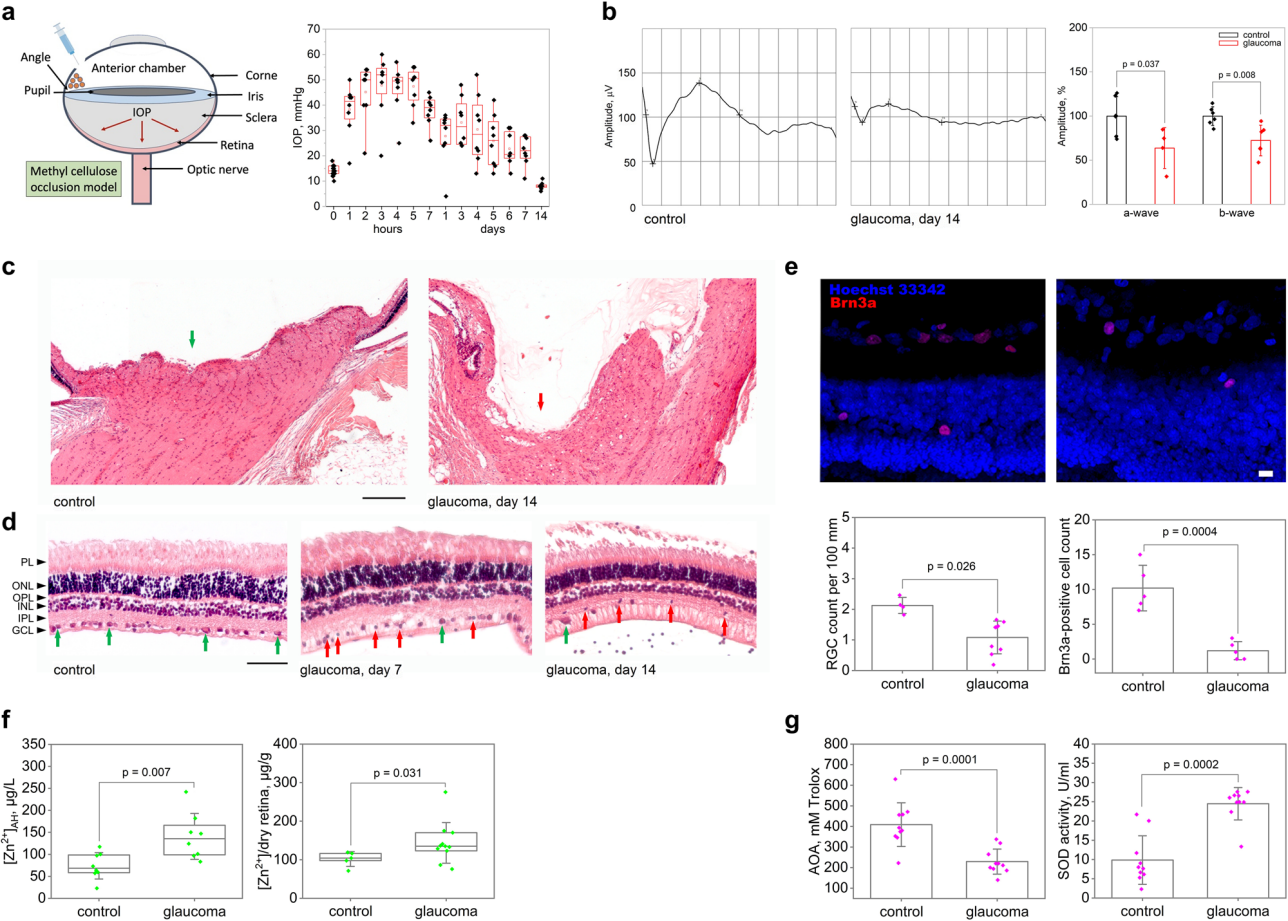
Glaucoma neuropathy induced by ocular hypertension in an animal model is associated with increased zinc content in AH and retina and oxidative stress. **a** Methylcellulose (2%) was injected into the anterior chamber of rabbits, resulting in sustained IOP elevation over 7–14 days (*n* = 8; *p* < 0.05 for all time points). **b** Representative electroretinogram and the mean amplitudes of its a- and b-waves showing a decrease in outer retinal activity on day 14 after methylcellulose injection (*n* = 6). **c** Representative micrographs of optic nerve cross-sections, hematoxylin and eosin, magnification 50×. Green and red arrows indicate normal optic disc of the control animal and optic disc excavation on day 14 after methylcellulose injection, respectively. Scale bar represents 200 µm. **d** Representative micrographs of retinal cross-sections, hematoxylin and eosin, magnification 200×. The designations are as follows: PL, photoreceptor layer, ONL, outer nuclear layer, OPL, outer plexiform layer, INL, inner nuclear layer, IPL, inner plexiform layer, and GCL, ganglion cell layer. Green arrows show viable RGC nuclei, red arrows show pyknotic (apoptotic) RGC nuclei on days 7 and 14 after methylcellulose injection. The scale bar represents 50 µm. **e** Representative confocal immunofluorescence images of retinal cross-sections obtained before and on day 14 after methylcellulose injection. The specific ganglion cell marker Brn3a^161^ was stained with polyclonal antibodies and detected using indirect fluorescence (red); call nuclei were visualized by Hoechst 3342 (blue). The bottom panel shows the results of morphometric evaluation of the total number of viable RGCs based on histologic (*n* = 9) and immunocytochemical (*n* = 5) analyses. Scale bar represents 10 µm. **f** Total zinc content in AH (*n* = 8) and retina (*n* = 11) of rabbits with ocular hypertension model was determined by AAS. Zinc concentration increased significantly on the 7th day after methylcellulose injection (*p* < 0.05). **g** Determination of oxidative stress markers in AH by colorimetric methods revealed a decrease in AOA and an increase in SOD activity on day 7 after methylcellulose administration (*n* = 10; *p* < 0.05). All data are presented as mean and error bars indicate SEM.

To verify whether the revealed increase in zinc and oxidative stress markers in AH of POAG patients was related specifically to glaucoma neuropathy (RGC death) caused by elevated IOP, and whether these conditions alter zinc homeostasis in the retina, we used animal (rabbit) model, where ocular hypertension was induced by obstructing the outflow of AH using injection of 2% methylcellulose into the anterior chamber (methylcellulose occlusion model)^17–19^. The injection reproducibly caused a rapid IOP increase in the rabbit eye from 10–20 to 50–70 mm Hg, which remained high for at least 7 h (Fig. 2a). The increase was associated with typical changes characteristic of glaucoma neuropathy caused by acute ocular hypertension^20^, including optic nerve excavation (Fig. 2c), a significant reduction of all wave amplitudes in ERG recordings (Fig. 2b), and death of retinal neurons (manifested as early as day 7) via the apoptotic pathway as evidenced by nuclear pyknosis (Fig. 2d). The most pronounced cell loss was observed in RGC layer (Fig. 2d, e), which corresponds to the damage of the human retina in glaucoma with IOP elevation. Overall, the model reliably reproduced glaucoma with IOP elevation, the main signs of which developed within 7-14 days after induction.

To evaluate whether the induced pathological process is associated with changes in zinc content in AH and the retina, the respective samples were collected from animals before or 7 days after the methylcellulose administration and analyzed by AAS (Fig. 2f). The mean zinc concentration in intact animals was determined as 74 ± 10 μg/L, which exceeded that for humans consistent with previous estimates^21–24^. At the same time, similarly to human patients, the animals with the ocular hypertension model showed an increase in zinc levels in AH reaching 141 ± 19 µg/L at day 7 after induction. Moreover, we observed a 40% increase in total zinc content in the retinas of these animals (102 ± 8 µg/g at day 0 versus 144 ± 16 µg/g at day 7). Notably, animals with the ocular hypertension model exhibited the same alterations in the antioxidant defense components as POAG patients (Fig. 2g). Thus, oxidative stress and increased zinc content in AH and retina are characteristic of early-stage neuropathy induced by ocular hypertension, suggesting that increase IOP could trigger the corresponding changes in patients with POAG.

### Increase in AH zinc in glaucoma can be recognized by signaling factor PEDF

**Fig. 3:**
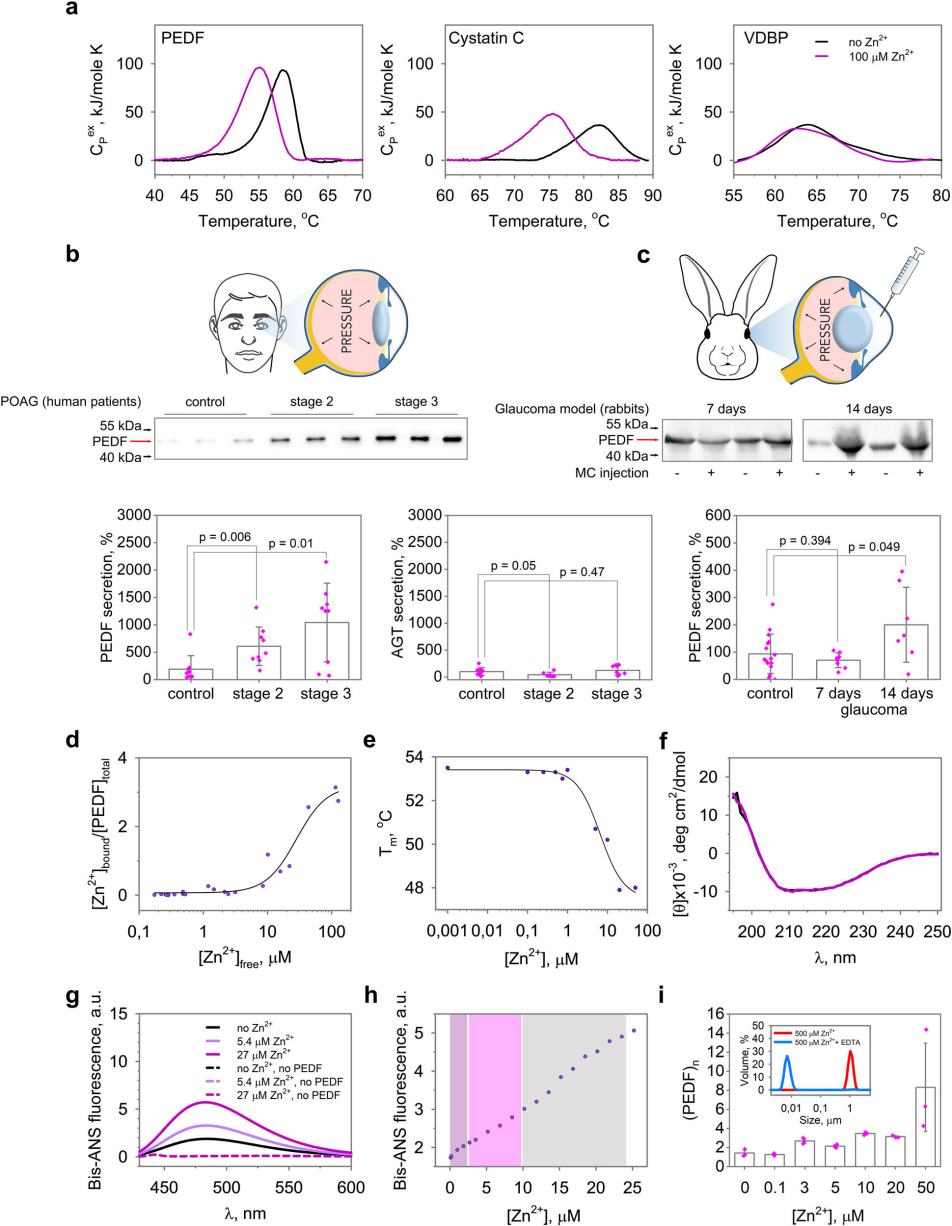
Ocular neurotrophic factor PEDF is zinc-dependent protein associated with glaucoma. **a** Zinc-binding properties of AH proteins PEDF, cystatin C, and VDBP, predicted to contain zinc-coordinating sites by ZincBindPredict, were probed by DSC. PEDF and Cystatin C exhibited a shift in melting temperature in the presence of Zn^2+^. **b** The content of signaling factors PEDF and AGT was analyzed in AH of POAG patients and control individuals (taken as 100%) by Western blotting. **c** PEDF content was determined in AH of animals with or without (taken as 100%) the ocular hypertension model on day 7 (*n* = 8) and day 14 (*n* = 8) after induction. A progressive increase in the amount of secreted PEDF was observed during development in both human and experimental glaucoma (*p* < 0.05). **d**, **e** Zinc binding to PEDF was investigated by equilibrium dialysis coupled with AAS and by recording the zinc-dependence of T_m_ using DSF, showing stoichiometry of 3 and micromolar affinity (solid curves represent the fit of the experimental data using the Hill equation). PEDF structure was analyzed using CD spectroscopy (**f**) and the fluorescent dye bis-ANS (**g**) in the presence of 5.4 µM or 27 µM Zn^2+^. Zinc binding did not affect the secondary structure but increased surface hydrophobicity of PEDF. **h** Effect of zinc on PEDF structure was monitored using bis-ANS fluorescence assay. Different zinc binding modes were observed at a Zn:PEDF ratio of 1:1 (magenta), at zinc excess 2-4 (pink), and at zinc excess >4 (light gray). **i** Oligomeric state of PEDF in the presence of zinc was evaluated by DLS. Zinc binding induced the transition of PEDF monomer into dimers (3-5 µM Zn^2+^), trimers (10–20 µM Zn^2+^) of larger oligomers (>20 µM Zn^2+^). In the presence of metal chelator EDTA, the oligomerization induced by high excess of zinc (500 µM) was reversed (inset), indicating its functional significance. The data in **b** and **i** are presented as mean and error bars indicate SEM.

If the increase in extracellular mobile zinc in glaucoma represents a cytotoxic signal^11^, it might be recognized by specific Zn^2+^-dependent proteins capable of mediating RGC death. Due to the vitreal-aqueous gradient, such proteins secreted in the retina accumulate in AH^9^. Therefore, to identify them, we searched for zinc-binding proteins in the core proteome of AH^25,26^, with a particular focus on signaling proteins showing changes in glaucoma. Preliminary identification and classification of Zn^2+^-binding sites were performed in proteins with resolved tertiary structure using machine learning-based ZincBindPredict program, which employs classifiers trained on ZincBind database to predict zinc coordination sites^27^. Among such proteins, 88.5% contained at least one potential Zn^2+^-binding site (Supplementary Data 10**;** see also Supplementary Data 11). The identified candidates included transport proteins, protease inhibitors, lipid-binding proteins, components of antioxidant defense, extracellular matrix and immune response proteins, and only two signaling factors, angiotensinogen (AGT) and pigment epithelium-derived factor (PEDF) (Table 2). The major part of the reveled proteins was previously demonstrated to exhibit Zn^2+^-binding properties confirming the accuracy of our predictions. Of the remaining three proteins, namely vitamin D-binding protein (VDBP), cystatin C and PEDF, were checked for zinc sensitivity in vitro using differential scanning calorimetry (DSC), which allows rapid preliminary screening of protein-cation complexation. VDBP did not possess zinc-binding properties, which was demonstrated by DSC (Fig. 3a) and confirmed using other physicochemical methods (Supplementary Fig. 2). In contrast, cystatin C and PEDF appeared to be able to coordinate zinc, as their thermodynamic properties changed significantly with increasing zinc concentration (Fig. 3a).

**Table 2:** Potential zinc-binding proteins of AH associated with POAG

Type	Gene	Protein	PDB	Predicted Zn^2+^-binding sites ^*a*^	Zn^2+^ binding according to (reference)		AH content according to (reference)		
					Y/N	K_D_, µM	%ALB^106^ ^b^	FC in glaucoma^148^	
								Min	Max
Transport	ALB	Serum albumin	7VR0	С2H1 – 6; С2H2 – 5; С3H1 – 1; D1H1 – 5; D1H2 – 1; E1H1 – 5	yes^149^	0.1^150^	100	0.94	1.49
	TF	Serotransferrin	7FFU	C2H1 – 2; D1H1 - 5; E1H2 – 1; E1H1 – 4	yes^149^	0.015^151^	18.21	0.37	1.27
	CP	Ceruloplasmin	1KCW	C2H1 – 1; D1H1– 18; D1H2 – 20; H3 – 9; E1H1 – 8; E1H2 – 11	yes^149^	3.3^152^	3.93	0.25	1.1
	TTR	Transthyretin	7Q3I	D1H1 – 2; D1H2 – 4; E1H2 – 1; E1H1 – 3	yes^149^	1^153^	3.72	0.95	2.08
	ORM1	Alpha-1-acid glycoprotein 1	3KQ0	D1H1 – 1	yes^149^	–	2.84	–	1.65
	GC	Vitamin D-binding protein	1KW2	D1H1 – 2; E1H1 – 2; E1H2 – 1	no	–	2.76	–	2.23
	HP	Haptoglobin	4X0L	6 D1H1. 2 D1H2. 2 E1H1	yes^154^	–	1.03	0.47	–
	HRG	Histidine-rich glycoprotein	4CCV	E1H1 – 1	yes^149^	–	0.71	0.39	1.59
Protease inhibition	SERPINA1	Alpha-1-antitrypsin	1KCT	D1H1 – 7; E1H1 – 3	yes^149^	–	5.55	0.89	2.11
	CST3	Cystatin C	3GAX	C2H1 – 2; D1H1 – 5; E1H1 – 1	no	–	1.97	0.07	4.6
	A2M	Alpha-2-macroglobulin	7VON	C2H1 – 2; D1H1 – 9; D1H2 – 2; E1H1 – 6	yes^149^	7.6^154^	1.73	0.34	–
	SERPINC1	Antithrombin-III	2B4X	D1H1 – 4	no	–	1.49	0.99	2.83
	SERPINA3	Alpha-1-antichymotrypsin	3DLW	D1H1 – 3	yes^149^	–	1.26	1.07	5.1
	SERPING1	Plasma protease C1 inhibitor	5DU3	C2H1 – 2; D1H1 – 8; E1H1 – 2	no	–	0.86	0.19	1.56
Lipid-binding	APOA1	Apolipoprotein A-I	1AV1	D1H1 – 2; D1H2 – 2; E1H1 – 6	yes^149^	–	2.3	0.37	1.79
	APOE	Apolipoprotein E	2L7B	D1H1 – 2	yes^149^	–	0.77	0.31	1.46
	AZGP1	Zinc-alpha-2-glycoprotein	1ZAG	C2H1 – 8; C2H2 – 4; D1H1 – 15; D1H2 – 5; E1H1 – 3	yes^155^	–	0.71	0.24	1.97
Antioxidant	GPX3	Glutathione peroxidase 3	2R37	D1H1 – 2	yes^156^	–	1.39	0.25	1.33
Hydrolases	RNASE1	Ribonuclease pancreatic	1H8X	С2Н1 – 1	yes^157^	–	0.7	0.79	1.02
ECM	CHI3L1	Chitinase-3-like protein 1	1HJV	D1H1 – 10; D1H2 – 3	yes^158^	–	0.48	0.64	1.87
Immunity	B2M	Beta-2-microglobulin	A16Z	C2H1 – 2; D1H1 – 20; D1H2 – 6; E1H1 – 8; E1H2 – 4	yes^159^	–	0.18	–	7.17
Signal transduction	SERPINF1	Pigment epithelium-derived factor	1IMW	D1H1 – 2; E1H1 – 1	no	–	3.36	–	– ^с^
	AGT	Angiotensinogen	2WXW	D1H1 – 8; D1H2 – 1; E1H1 – 5	yes^149^	–	0.62	–	9.31^c^

^a^Zn^2+^-binding sites predicted using ZincBindPredict program with probability > 0.95 are presented (see Supplementary Data 10, 11); zinc-coordinating residues (cysteine (C), histidine (H), aspartic acid (D), glutamic acid (E)), their number in each site, and the number of sites are indicated.

^b^Albumin concentration in AH is 4.6 µM^160^.

^c^The results of this study.

The above analysis identified two signal transduction proteins, AGT and PEDF, that could transmit cytotoxic zinc signals. If they are involved in POAG, one should expect increased secretion of these proteins to be reflected in AH content. Analysis of AH samples from the cohort of POAG patients (Supplementary Data 1) showed that AGT concentration did not change in glaucoma, whereas PEDF content increased dramatically with POAG progression, reaching approximately 10-fold excess at stage 3 of the disease (Fig. 3b). Moreover, a similar trend was observed in rabbits on day 14 of experimental glaucoma (Fig. 3c).

Overall, PEDF was identified as a unique POAG-associated zinc-dependent signaling protein. It should be emphasized that PEDF functions through activation of cell surface receptors^28^ and, therefore, can directly receive and transduce signals of increased mobile zinc in POAG. Moreover, given that PEDF is a key neurotrophic factor in the retina^29–31^, the effect of zinc on its activity may influence the defense mechanisms and/or death pathways regulating cell fate in this disease.

### Zinc binding to neurotrophic factor PEDF determines its oligomeric state

To assess whether PEDF would respond to the identified changes in mobile zinc associated with POAG (from 0.5 to 1–7 μM, see Fig. 1a) and how these conditions would affect its activity, we analyzed its affinity for Zn^2+^ and the effect of zinc on structural properties of the protein in vitro. Monitoring zinc binding to PEDF using equilibrium microdialysis in combination with AAS showed a stoichiometry of 3 and half-maximal binding at 28 μM [Zn^2+^]_free_ (Fig. 3d). In turn, a nanoDSF study monitoring zinc coordination by decrease in PEDF melting temperature T_m_ showed half-maximal zinc binding at 6.6 μM **(**Fig. 3e**)**. Interaction with zinc did not affect the secondary structure of PEDF, as demonstrated by far-UV CD spectroscopy, but increased accessibility of its surface hydrophobic sites, as determined using fluorescent probe bis-ANS **(**Fig. 3, f, g**)**. The results of PEDF titration with zinc in the presence of bis-ANS indicated several modes of zinc binding, observed at molar excess of zinc over PEDF of 1, 2-4 (high-affinity Zn^2+^ binding) and above 4 (low-affinity Zn^2+^ binding) **(**Fig. 3h**)**. From these data, the apparent dissociation constant for the primary Zn^2+^-binding site of PEDF was estimated as 4 μM **(**Supplementary Fig. 3**)**. According to DLS experiments, zinc binding led to the transition of PEDF into dimers (3-5 µM Zn^2+^), trimers (10-20 µM Zn^2+^) of larger oligomers (>20 µM Zn^2+^) **(**Fig. 3i**)**. Importantly, addition of zinc chelator led to dissociation of PEDF oligomers that were formed even at high zinc excess **(**Fig. 3i, inset**)**, indicating that the protein undergoes functional oligomerization.

Taken together, these data suggest that PEDF is able to recognize changes in mobile zinc characteristic for glaucoma, responding by reversible oligomerization.

### Crystal structure of PEDF reveals five types of zinc binding sites

**Fig. 4:**
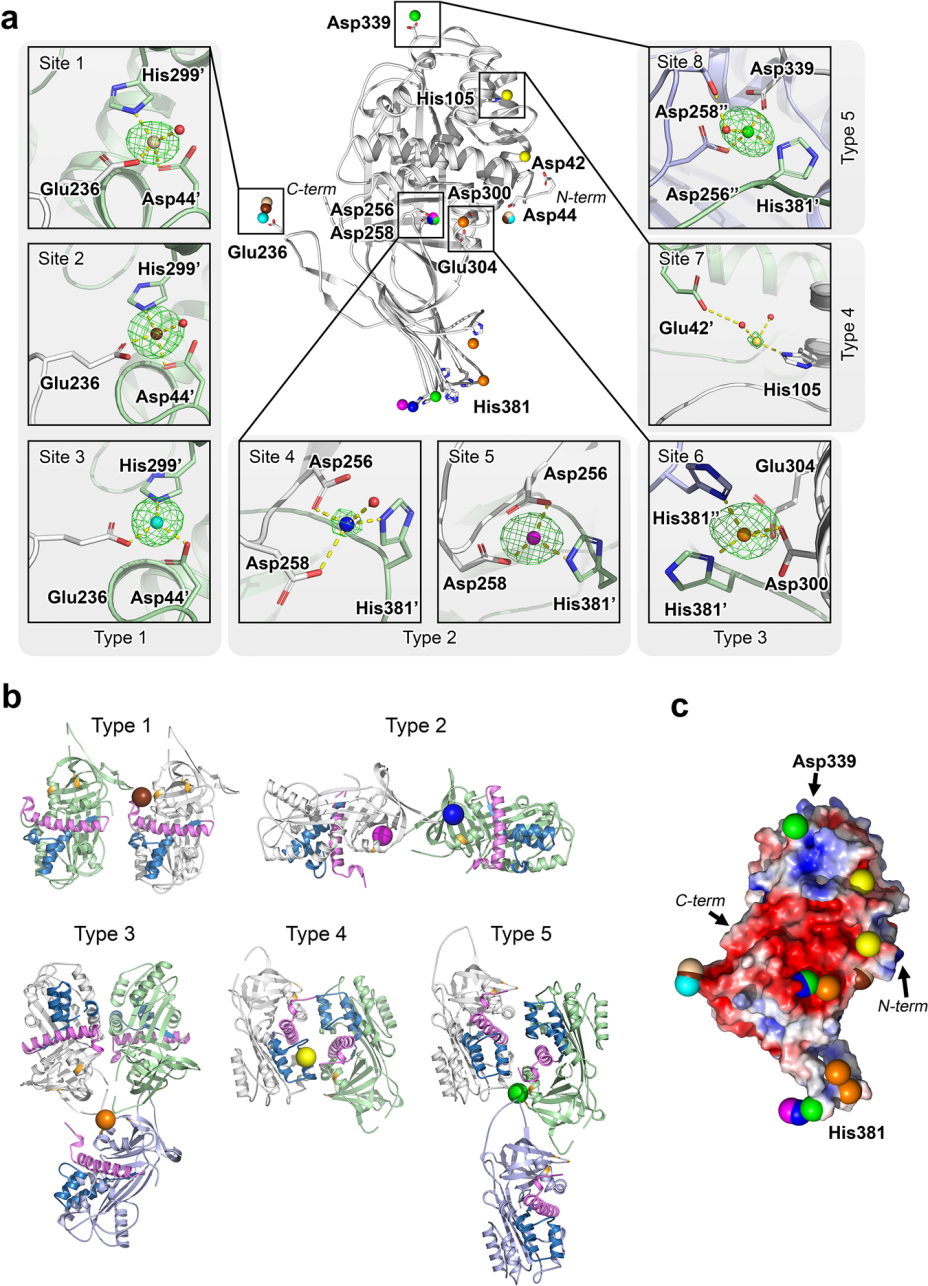
Crystal structure of PEDF revealed zinc binding sites. **a** The crystal structure of PEDF revealed 8 zinc coordination sites located at the intermolecular interface of PEDF dimers and trimers. In the figure, the sites are numbered clockwise and divided into 5 types according to their coordination. Zinc ions corresponding to sites 1–8 are shown in wheat, chocolate, blue, blue, purple, orange, yellow and green colors, respectively. Site 1 was observed in P2_1_2_1_2 crystals, while sites 2-8 were found in P2_1_2_1_2_1_ crystals. Anomalous difference maps are indicated as green mesh at the 7σ-level. Ions of different colors appeared several times in the figure, showing different interaction interfaces on different monomers within one oligomer. Different colors of protein molecules correspond to different molecules in one oligomer. Zinc coordination bonds are represented by yellow dashed lines. Refer to Supplementary Fig. 9 for additional structural details of the Zn^2+^-binding sites. **b** The filling of five types of Zn^2+^-binding sites leads to the formation of five variants of PEDF dimers and trimers, which affects the accessibility of target recognition sites important for PEDF functions. These include PEDF-R binding and neurotrophic activity (44-mer fragment 78-121, shown in blue), anti-angiogenic activity (34-mer fragment 44-77, shown in purple), and collagen binding (Asp256, Asp258, and Asp300 shown in orange). Refer to Table 3 for details of function inhibition resulting from PEDF di-/tri-merization. **c** Zinc coordination alters the surface properties of PEDF, such as weakening the negative electrostatic charge (red) of the characteristic region of the protein.

Analysis using ZincBindPredict revealed only two potential Zn^2+^-binding sites in PEDF molecule. To identify all the zinc coordination sites, we next crystallized PEDF in the presence of this metal and collected X-ray diffraction data. Crystals were obtained in two space groups, P2_1_2_1_2 and P2_1_2_1_2_1_, with one and five protein molecules in the asymmetric unit. In both cases, the crystals gave diffraction data up to ~2 Å. Zinc binding sites were identified in both space groups: 1 site for P2_1_2_1_2 and 7 sites for P2_1_2_1_2_1_ (Fig. 4a). Importantly, all identified sites were located at the intermolecular interfaces and included coordinators from two or three protein molecules (Fig. 4b) confirming the role of zinc as a promoter of PEDF oligomerization observed in DLS studies (**see** Fig. 3i). All 8 binding sites can be classified into 5 types based on their coordination partners **(**Table 3**)**: three “type 1” sites (Asp44, His299 from protein molecule 1, Glu236 from protein molecule 2 and a water molecule), two “type 2” sites (Asp256, Asp258 from molecule 1, His381 from molecule 2 and a water molecule), “type 3” site (His381 from molecule 1 and molecule 2, and Asp304, Asp300 form molecule 3), “type 4” (His105 from protein molecule 1 and two water molecules, one of which is H-bonded to Glu42 of the protein molecule 2) and “type 5” site (Asp256 from molecule 1, His381 residues from molecule 2, Asp339 form molecule 3, and a water molecule). Two of the binging sites (#4 and #7) have lower occupancy, which might correlate with their affinity for zinc. The type 1 site, which was also predicted by ZincBind appears to be the strongest and most important, and its occupancy seems to affect the properties of PEDF at low zinc concentrations (see Fig. 3, h, i). Considering the incomplete occupancy of binding sites in PEDF crystal (Table 3), the described configuration of zinc binding is consistent with the average stoichiometry of 3–4 observed in biophysical studies (see Fig. 3d, h). Notably, zinc coordination did not significantly change the secondary and tertiary structure of PEDF: the RMSD Cα in secondary structure elements between the current structures and the previously resolved structure of PEDF without zinc (PDB ID 1IMV) is below 0.5 Å, which is in accord with the results of CD spectroscopy measurements (see Fig. 3f). However, zinc affected the surface properties of PEDF, since some of the identified sites are located near an intensely negatively charged region of the protein, and the appearance of a positively charged ion in this region partially neutralizes the negative surface charge (Fig. 4c). This phenomenon explains the facilitation of bis-ANS binding (see Fig. 3g, h) and together with changes in the quaternary structure of PEDF, suggests alterations in the functional properties of PEDF in the presence of zinc.

**Table 3:** Zinc-binding sites in PEDF identified by X-ray crystallography

Site number	Site type	Zinc coordinators				PDB ID	Occupancy	Affected activity ^a^
		Molecule 1	Molecule 2	Molecule 3	H_2_O			
1	1	Asp44, His299	Glu236	-	+	9J3Q	0.90	Neurotrophic, anti-angiogenic, ECM binding
2	1	Asp44, His299	Glu236	-	+	9J3P	0.90	Neurotrophic, anti-angiogenic, ECM binding
3	1	Asp44, His299	Glu236	-	+	9J3P	0.90	Neurotrophic, anti-angiogenic, ECM binding
4	2	Asp256, Asp258	His381	-	+	9J3P	0.60	ECM binding
5	2	Asp256, Asp258	His381	-	+	9J3P	0.90	ECM binding
6	3	His381	His381	Asp304, Asp300	-	9J3P	0.90	ECM binding
7	4	His105	Glu42 (H_2_O)	-	+	9J3P	0.40	Neurotrophic, anti-angiogenic, ECM binding
8	5	Asp256, Asp258 (H_2_O)	His381	Asp339	-	9J3P	0.90	ECM binding, neurotrophic, anti-angiogenic

^a^Suppression of PEDF functions associated with zinc binding and impeded access to sites responsible for neurotrophic (44-mer fragment 78–121)^61,73–75^, antiangiogenic (34-mer fragment 44-77)^85^, or ECM-binding (Asp256, Asp258 and Asp300)^86^ activities.

Overall, zinc binds to at least three high-affinity intermolecular sites in PEDF, and this binding leads to a decrease in the negative surface charge and promotes oligomerization of the protein, which might reflect on its ability to interact with molecular targets.

### PEDF secretion by retinal cells is enhanced under zinc stress conditions

**Fig. 5:**
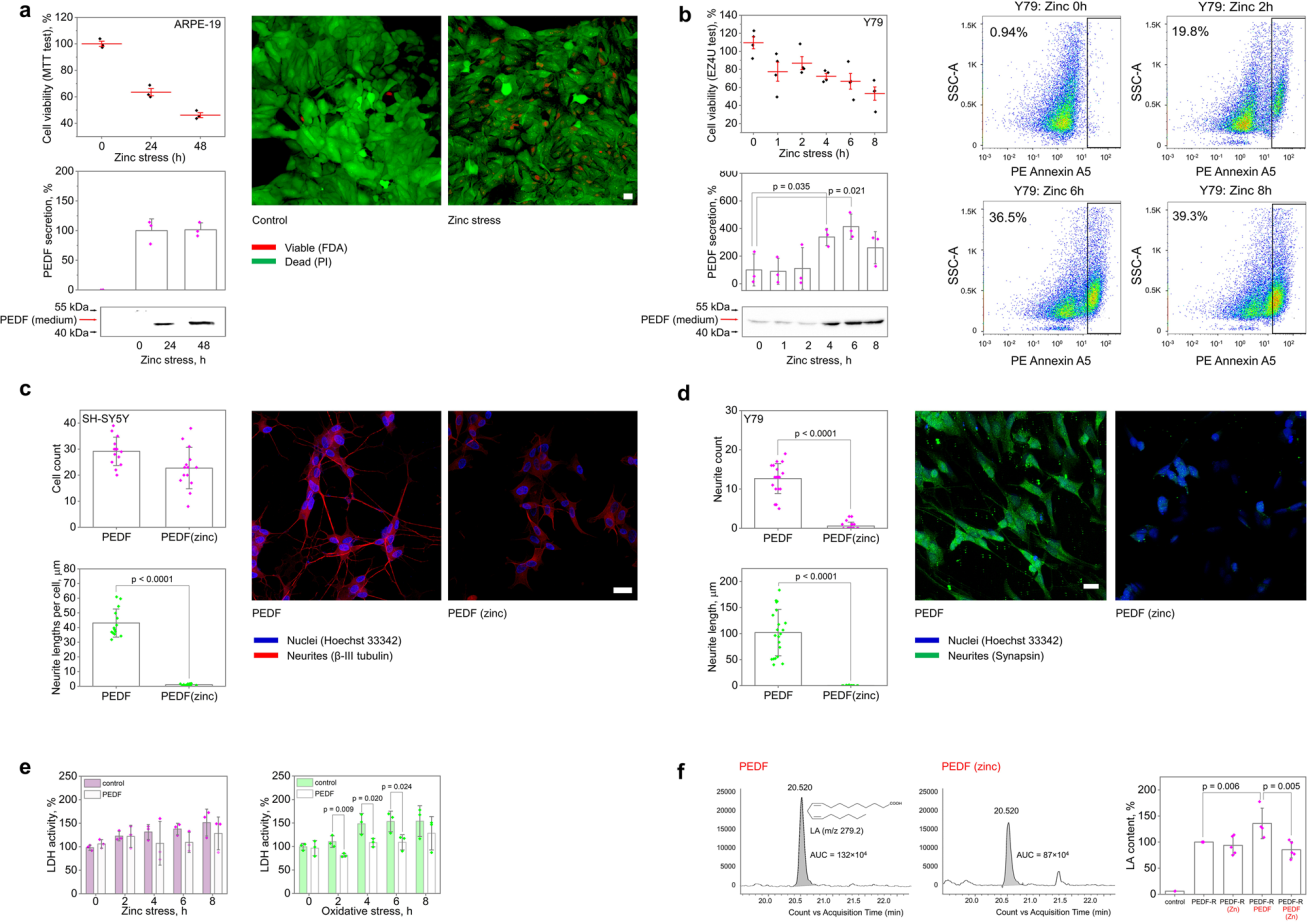
Zinc stimulates secretion but suppresses neurotrophic activity of PEDF. Effect of zinc stress on PEDF secretion was studied in ARPE−19 (**a**) and Y79 (**b**) cells. **a** APRE−19 shows a slow response to zinc stress (half-maximum after 24 h), as confirmed by MTT test, as well as fluorescein diacetate/propidium iodide (FDA/PI) viability assay and confocal microscopy (Scale bar represents 25 μm, *n* = 3). **b** Zinc treatment decreased viability (half-maximal after 8 h) and induced apoptosis of Y79 cells, as evidenced by EZ4U test and flow cytometric annexin A5 assay (*n* = 6). In both cases, zinc stress was accompanied by PEDF release (*p* < 0.05). The amount of PEDF in the absence (Y79) or at 24 h (ARPE−19) of stress was taken as 100%. **c-e** Effect of zinc on the neurotrophic activity of PEDF was studied on SH-SY5Y cells (**c**) and Y79 **c**ells (**d-e**). **c** Preincubation of PEDF with zinc reduced its axogenic activity in respect to differentiated SH-SY5Y cells without affecting cell count (Scale bar represents 25 μm, *n* = 14). **d** Zinc inhibited the ability of PEDF to stimulate neuronal differentiation of Y79 cells (Scale bar represents 10 μm, *n* = 21). Neurites and nuclei were stained with anti-β-III tubulin (**c**) or synapsin (**d**) antibodies and Hoechst 33342, respectively, and visualized by confocal microscopy. **e** PEDF att**e**nuated Y79 cell death under oxidative stress but not zinc stress, as shown by LDH assay (*n* = 3). Leaked LDH activity in the absence of stress was taken as 100%. **f** Phospholipase activity of PEDF-R in ARPE−19 cell membranes was monitored in the presence of apo or zinc-bound PEDF by quantifying fatty acid release from model phospholipid substrate using HPLC-MS/MS. Extracted ion chromatogram illustrating differences in linoleic acid ([M-H]^-^ LA, m/z 279.2) formation in the presence of apo and zinc-bound PEDF. The histogram on the right illustrates the attenuation of PEDF-mediated enhancement of PEDF-R activity in the presence of zinc (*p* < 0.05). PEDF-R activity without PEDF/zinc was taken as 100%. All data are presented as mean and error bars indicate SEM.

PEDF is secreted in the retina in response to various types of stress, performing a neuroprotective function^32^. Given that elevated levels of extracellular PEDF and zinc have been identified here as associated events in glaucoma, we tested whether PEDF could be secreted directly by retinal cells in response to extracellular zinc stress. To this end, we induced the cytotoxic effects by 100 µM Zn^2+^^33^ in two cell lines, ARPE−19 and Y79, representing models of RPE and retinal neurons, respectively. ARPE−19 cells were relatively resistant to zinc toxicity: a half-maximal effect was observed only 48 h after exposure to excess zinc (Fig. 5a). In the case of Y79, incubation with zinc decreased cell viability, reaching a half-maximal effect (50% of remaining live cells) at the eighth hour. The zinc-induced cell death occurred by apoptosis, as indicated by annexin A5 staining (Fig. 5b). Despite these differences, exposure of the both cell lines to zinc stress markedly stimulated accumulation of PEDF in the culture medium, which was low (Y79) or completely absent (ARPE19) under normal conditions. The observed phenomenon can be attributed to active secretion rather than protein leakage from dying cells, as it was accompanied by a decrease in the content of PEDF in the remaining intact cells (Supplementary Fig. 4).

In summary, PEDF secretion and accumulation in AH, which are elevated in glaucoma, may be directly stimulated by an increase in extracellular zinc and accompany Zn^2+^-induced apoptosis of retinal cells.

### Zinc binding inhibits the neurotrophic activity of PEDF

PEDF release stimulated by zinc in glaucoma may lead to its colocalization and complexation, which may affect the neurotrophic function of the factor. To check out this scenario, we analyzed the effect of zinc on axogenic and differentiative activity of recombinant PEDF under normal conditions and its prosurvival activity under stress conditions in appropriate cellular models, human neuroblastoma SH-SY5Y cells and Y79 cells^34–36^. Neither apo-PEDF nor Zn^2+^-saturated PEDF had a significant effect on the normal viability of SH-SY5Y cells (Fig. 5с), indicating that in the latter case zinc remained bound to the protein as it would otherwise exert cytotoxic action (Supplementary Fig. 5). At the same time, SH-SY5Y cells treated with Zn^2+^-saturated PEDF exhibited 43-fold less total neurite lengths than apo-PEDF-treated cells, indicating that zinc binding suppresses the axogenic activity of the factor (Fig. 5c). In the case of Y79 cells, the presence of zinc at a non-cytotoxic concentration (1 μM) almost completely suppressed the ability of PEDF to stimulate neuronal differentiation (Fig. 5d).

**Fig. 6:**
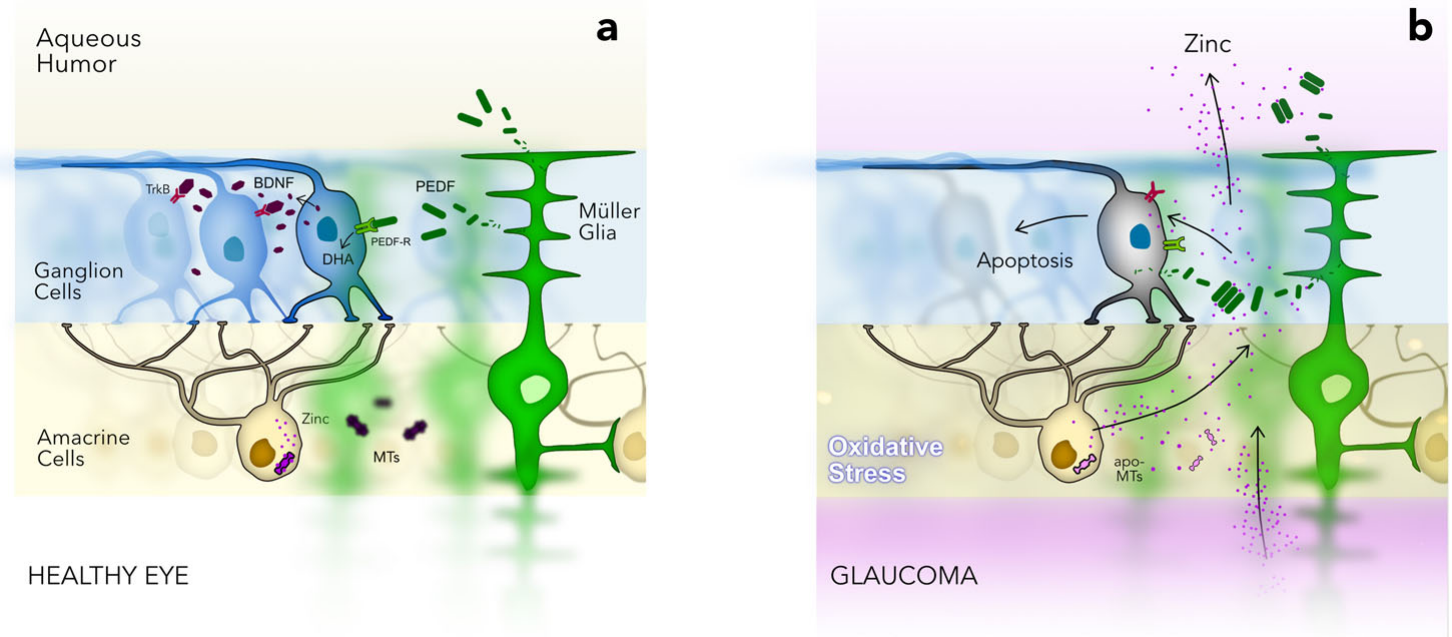
Blockade of PEDF by zinc may contribute to neurodegeneration in glaucoma. **a** In the healthy retina, PEDF is a component of normal trophic support of RGCs that is secreted by Müller cells and stimulates phospholipase A2 activity of PEDF-R, resulting in increased production of DHA and upregulation of other neurotrophic factors such as BDNF. **b** In glaucoma, oxidative stress causes the release of mobile zinc from metallothioneins (MTs) into the extracellular space, which triggers apoptotic signaling in RGCs. Under these conditions, PEDF is intensely secreted by retinal cells but becomes inactive because zinc binding reduces its negative surface charge and promotes oligomerization, which impairs the neuroprotective and regenerative activity of the protein. Along with suppression of other Zn^2+^-sensitive neurotrophic factors (BDNF, NGF, etc.) that support RGCs growth and survival, this mechanism contributes to neurodegeneration in glaucoma.

The effect of zinc on the neuroprotective function of PEDF was evaluated on Y79 cells. Direct measurement of such activity under zinc stress was hindered because of the inability to separate the effect of free Zn^2+^ on the protein and on the targeted cells. Therefore, we compared cytoprotective activity of PEDF under two types of stress, zinc stress (mimicking the neurotrophic activity of the protein in complex with Zn^2+^) and oxidative stress (mimicking neurotrophic activity of the apo-protein). The concentration of recombinant PEDF was 1 μM, which corresponds to its maximal content in AH of patients with POAG (baseline concentration of 0.1 μM^37^ increased 10-fold, see Fig. 3b). The conditions were selected so that after 8 h of incubation, the residual number of live cells was approximately 50% in both cases. Treatment of Y79 cells with PEDF added to the cultural medium attenuated H_2_O_2_-induced cell death at least during 6 h of the exposure (Fig. 6e). Meanwhile, the suppressive effect of PEDF on zinc toxicity under the same conditions was almost absent, suggesting that zinc inhibits the protective action of the protein.

Finally, we investigated whether the observed effects of zinc were due to its direct influence on PEDF ability to activate its specific receptor PEDF-R (patatin-like phospholipase domain-containing protein 2, PNPLA2)^30^. To this end, the phospholipase activity of the receptor embedded in ARPE−19 cell membranes toward a model linoleic acid (LA)-containing phospholipid substrate was monitored in the presence of apo or Zn^2+^-saturated PEDF by quantifying LA release using a targeted HPLC-MS/MS assay (Fig. 5f). The presence of zinc alone had not effect on PEDF-R. Addition of PEDF without zinc led to stimulation of receptor activity by about 40%. At the same time, pre-incubation of PEDF with zinc completely canceled its effect. Thus, given that zinc changes charge properties and quaternary structure of PEDF (see Figs. 3g, 4b, c), the resulting oligomer of the protein appears to be unable to bind and activate PEDF-R.

Overall, PEDF turned out to be one of the most relevant extracellular targets of mobile zinc, as its level is elevated in zinc-enriched glaucomatous AH, and its secretion from RPE and retina-like cells is stimulated by zinc and accompanies their Zn^2+^-induced apoptosis. All these conditions lead to the transfer of secreted PEDF to a complex with zinc, rendering it unable to activate PEDF-R and negatively affecting its neurotrophic activity, which may contribute to retinal neurodegeneration.

## Discussion

We have shown that the progression of POAG is associated with a marked increase in the level of zinc released in the ocular system and accumulated in the AH, reaching 2–15-fold above baseline concentrations in some patients. These data are in general agreement with recent estimates^23,38^, but our study is the first in which AH zinc was measured as a function of POAG stage and shown to be elevated already in the early stages of the disease. The retina contains large amounts of zinc and makes the most significant contribution to its ocular homeostasis^4^. Indeed, our measurements indicate that total zinc content in the rabbit retina is about 50-fold higher than in AH (20 ng versus 1000 ng). Moreover, our data suggest that the early stages of glaucoma neuropathy induced by elevated IOP in rabbits are associated with an increase in total zinc, not only in AH but also in the retina. One source of this increase may be the RPE/choroid, which contains an even higher (2.4-fold) amount of zinc than retina^39^ and has a nourishing function in respect to the neuroretina and a close interrelationship with it in terms of initiation and propagation of oxidative stress^40,41^. The vitreal-aqueous gradient allows retinal secreted compounds to accumulate in AH^9,13^. We suggest that, following this gradient, zinc complexes with proteins or other compounds from the retina may undergo anterior diffusion. Thus, the anterior transport of retinal zinc released in glaucoma can result in an increase in its concentration in AH observed in our experiments.

What are the mechanisms underlying the elevation of zinc levels in POAG? During ischemia, zinc can be released in the retinal cells as a result of dissociation from metallothioneins and other proteins and transfer to extracellular space via ZnT transporters, as well as by leakage from dying cells^4,12,42^. Consistently, under conditions of oxidative stress, chelatable zinc is liberated in RPE cells^4^. According to the results of animal studies, retinal amacrine cells secrete zinc in response to optic nerve injury^11,12^. A similar increase in extracellular cytotoxic zinc has been demonstrated, for instance, in a rat model of transient global ischemia, where it induces selective and delayed degeneration of hippocampal CA1 pyramidal neurons^43^. Elevated zinc secretion by glycinergic neurons is a general characteristic of excitotoxicity^5,7,44^, one of the critical factors in the development of glaucoma^8^. In our study, the increased zinc in AH was observed in an animal model of ocular hypertension, where it specifically accompanied the neuropathy induced by IOP elevation, the most common driving force of POAG. The hallmark of this condition is chronic retinal ischemia-reoxygenation and oxidative stress^8,45^, which can induce the release of zinc in the retina from oxidized proteins^46,47^. Consistently, we recorded signs of oxidative stress in AH, both in POAG patients and animals with ocular hypertension, which included decreased AOA and increased in Zn^2+^-dependent antioxidant enzyme SOD, in agreement with previous findings^48–50^. Interestingly, extracellular SOD is a global indicator of zinc status in humans: in serum its activity correlates with zinc concentrations and increases upon zinc supplementation^51,52^, and a similar trend seems to be characteristic of AH. Overall, zinc release may accompany the main pathogenetic processes in glaucoma, thus being one of the important elements of the disease.

What can be the pattern of low molecular weight chelators carrying excess zinc in POAG? Apparently, the intraocular flux of at least some metabolites follows the same vitreal-aqueous gradient (see above), allowing the composition of retinal zinc-chelate complexes to be assessed by metabolomic analysis of AH. For example, amino acids are actively transported from the vitreous body to the anterior chamber of the eye^53^. In our study, more than half of AH metabolites showing changes in glaucoma were recognized as zinc chelators. Among the zinc-binding molecules with the highest affinity, the general trend was a decrease in citric acid and an increase in amino acids, such as cysteine. Thus, POAG progression seems to be associated with the accumulation of zinc-amino acid complexes, especially in the early stages of the disease. The most probable effect of such redistribution of zinc is the enhancement of its cellular uptake, since amino acids act as zinc ionophores, being rapidly taken up by cells via specific SLC transporters^54^. Among the remaining metabolites, particularly notable are components of purine metabolism, ribose-5-phosphate, and uric acid, demonstrating approximately 4-fold elevation in POAG. Uric acid is one of the major constituents of AH (0.1 mM), and its increase in glaucoma was observed in other experimental cohorts^55^. Interestingly, the blood levels of uric acid are related to zinc content: zinc diet increases its synthesis^56^. Depending on conditions, uric acid may act as an antioxidant or pro-oxidant^57^, and its rise in AH may therefore be another indication of oxidative stress. Importantly, most of the identified chelators are present in AH at high concentrations but have relatively low affinity for Zn^2+^ (10^−3^–10^−6^ M) and thus form a pool of “loosely bound” zinc^3–5^ available to proteins with similar or higher affinity to mediate its physiological and/or pathological activity.

What is the nature of these proteins? Our analysis revealed that 75-90% of the AH core proteome contains potential Zn^2+^-binding sites, suggesting an important role of secreted zinc in the ocular system. Shortlisted candidates (Table 2) include mostly well-known zinc transport proteins. Novel and most probable targets of the excessive zinc in glaucoma are signaling factors, which may recognize zinc signals and directly trigger RGC death pathways. Our prediction revealed only two candidates with signaling activity (Table 2), among which the most relevant is PEDF exhibiting dramatically increased (5–10-fold) secretion in glaucoma. Interestingly, PEDF belongs to the serpins (SERPINF1), a family of serine proteinase inhibitors other members of which have also been identified as zinc-binding proteins (Table 2), suggesting that zinc coordination is a common property of this family and may affect ocular proteostasis. However, PEDF lacks a consensus sequence for the serpin reactive center region and exhibits no antiprotease activity, while being a major neurotrophic factor in the retina that promotes neurogenesis and enhances the viability of photoreceptors, amacrine cells, and other neurons^58^. Being produced primarily by RPE and Müller glia^36,59,60^, PEDF is at the same time the sixth most abundant component of AH^26^ with a concentration of about ~0.02–0.1 μM under normal conditions^61,62^, which, according to our data, increases 5–10 fold in glaucoma (Fig. 3b).

Given that the baseline concentration of PEDF in the retina is 25-fold higher than in AH^63^, it is likely to undergo anterior diffusion along the vitreal-water gradient, as previously shown in the case of VEGF or IL-6^64–67^. PEDF acts through the interaction with cell surface receptors, mainly PEDF-R, which contribute significantly to the survival of retinal cells^68,69^. The binding of PEDF stimulates phospholipase A2 activity of PEDF-R and the production and release of DHA, the most represented fatty acids of the retinal membranes^70,71^. DHA is the precursor of neuroprotectin Dl, a signaling lipid playing a neuroprotector role in the retina and RPE^72^. Moreover, DHA and neuroprotectin Dl induce expression of other neurotrophic factors, BDNF (brain-derived neurotrophic factor) and NGF (nerve growth factor)^71^. As a result, PEDF prevents apoptosis of retinal neurons by decreasing intracellular calcium and upregulating antiapoptotic factors^73^. PEDF alterations are associated with various retinal diseases, including AMD and diabetic retinopathy^74,75^, and growing evidence points on its neuroprotective effect in glaucoma. Indeed, in mice, elevated IOP stimulates the expression of PEDF and PEDF-R in RGCs and Müller cells, and inhibition of PEDF signaling promotes pressure-induced apoptosis of RGCs, which can be abolished by recombinant PEDF^30^. In addition, activated PEDF signaling can directly suppress apoptosis in cultured adult rat RGCs^76,77^. In glaucomatous retina, PEDF exerts neuroprotective effects on RGC with high expression of PEDF-R^30,78^. Moreover, due to its potent activity, PEDF gene transfer was suggested as a neuroprotective therapy in glaucoma^79^.

Our data demonstrate that PEDF is indeed intensely secreted in POAG, accumulating in AH of patients, and this secretion increases as the disease progresses and accompanies elevation of zinc levels in both AH and retina. This effect is specific to glaucoma, as AH levels of this factor are usually conversely reduced with normal aging and common ocular conditions such as myopia^61,80^. Consistently, in RPE and retinal cell models, PEDF secretion is stimulated by extracellular zinc and is associated with Zn^2+^-induced apoptosis (Fig. 5a, b). The exact pathway governing this secretion is unknown, but one can assume, for example, the involvement of GPR39, a G protein-coupled receptor using zinc as an agonist, the activation of which induces PEDF release^81,82^. This mechanism may originally be intended to protect and regenerate retinal cells, but it becomes compromised by excess zinc, which, according to our in vitro data, binds to PEDF and alters its structural and functional properties. Zinc concentration corresponding to filling the high-affinity site in PEDF (4 μM) matches the level of elevated zinc in AH of some patients with POAG (2–7 μM), suggesting that this metal is unlikely to bind to PEDF under normal conditions (0.4–0.5 μM Zn^2+^) but may be captured under pathologic conditions. We estimated that the total zinc content in the retina is 50 times higher than in AH and increases in glaucoma (see above), i.e., the probability that extracellular zinc reaches the concentration required for PEDF saturation is even higher. Zinc binding causes reversible dimerization/trimerization of PEDF, which may negatively affect its interaction with PEDF-R and inhibit its neurotrophic activity. Indeed, PEDF-R binding and neurotrophic activity of PEDF have been shown to be provided by its 44-mer fragment spanning amino acid residues at positions 78–121^70,83–85^. According to our structural analysis, zinc binding to sites of types 1, 4 and 5 **(**Table 3**)** results in this fragment localizing at the intermolecular interface of the resulting dimer/trimer, impeding its access to the receptor (Fig. 4b). Consistently, in our cellular models, zinc diminished ability of the protein to activate PEDF-R and suppressed its differentiative, axogenic and prosurvival activities (Fig. 5c–f). Overall, in glaucoma, we can suggest two opposite effects, namely stimulation of PEDF secretion in response to zinc stress and its inactivation by zinc (Fig. 6).

It should be emphasized that zinc binding to type 1 and type 5 sites also affects access to the 34-mer fragment 44-77 (contains Zn^2+^-coordinating Asp44), another crucial site of PEDF responsible for its anti-angiogenic activity^85^ (Fig. 4b). The interaction with this fragment can be further influenced by the occupancy of the low-affinity type 4 site **(**Table 3), as it is located in the contacting region and contains Glu42 holding one of the zinc-coordinating water molecules. Filling the type 1 site may also affect PEDF binding to type I collagen as it would reduce the negative surface charge of Asp300, a key residue crucial for collagen recognition^86^ that is adjacent to the Zn^2+^-coordinating His299 (Fig. 4a). In addition, collagen recognition may be affected by the occupancy of site #4, as in this case the dimerization interface prevents access to the collagen binding site. However, complete blockage of ECM attachment can be expected upon filling of type 2, 3 and 5 sites, as they contain Asp256 and Asp258, or Asp 300, which are directly involved in collagen binding^86^. Notably, the residues forming type 1 and type 5 sites in PEDF are highly conserved among mammalians, whereas the key histidine ensuring coordination in type 2, 3, and 5 sites appears to be specific to the human ortholog (Supplementary Fig. 6). Overall, simultaneous or sequential filling of zinc-binding sites appears to suppress the full range of PEDF functions in the case of the human protein and at least its neurotrophic and antiangiogenic activities in the case of other orthologs.

Interestingly, a functionally similar mechanism has been previously suggested for neurotrophins (BDNF, NGF, and NT3): all these factors are zinc-binding proteins and undergo Zn^2+^-induced deactivating conformational changes that in the case of BDNF, prevent it from interacting with its receptor TrkB^87,88^. Through selective inhibition of BDNF activity, zinc, for instance, causes motor neuron death in amyotrophic lateral sclerosis^89^. Thus, we can propose that negative regulation by zinc is a common feature of neurotropic factors of different classes and may represent a common mechanism for many neurodegenerative diseases. The molecular mechanisms of RGC death followed by axonal degeneration in glaucoma are not fully understood, but one generally accepted possibility is that apoptotic signaling is triggered under conditions of impaired trophic support due to a blockade of retrograde axonal transport of BDNF/TrkB^90,91^. In response to injury, RGCs upregulate BDNF expression, thereby protecting the remaining cellular population in an autocrine manner, and RGCs survival can also be stimulated by the administration of exogenous BDNF^91,92^. Consistently, the AH level of BDNF declined in patients with early POAG and increased in developed disease^93^. TrkB is expressed not only in RGCs, but also in amacrine cells and Müller glia, indicating their sensitivity to BDNF action^91^. Stimulation of glial TrkB by BDNF increases secretion of a number of neurotrophic factors, including neurotrophins, BDNF, and NGF, which enhance RGC viability in a paracrine manner^92^. Similar effect is produced by PEDF via PEDF-R/DHA pathway^71^. Thus, inhibition of the activity of all these factors by secreted zinc seems to be one of the key nodes of glaucoma pathogenesis (Fig. 6), and its targeting may represent a promising option in POAG treatment.

It should be added that PEDF/neurotropic factors are unlikely to be exclusive targets of excess extracellular zinc in glaucoma, which can affect a number of proteins (Table 2). In this work, we focused only on signaling proteins/cascades as the most probable mediators of life and death decisions in retinal cells. Further studies are required to explore the full range of processes impacted by mobile zinc in order to understand the complex pathogenesis of the disease and to develop specific therapies.

## Methods

### Characteristics of human participants

The study involved 57 patients who underwent surgical treatment at the Helmholtz National Medical Research Center for Eye Diseases. The control group included patients without visual pathology, except for cataract. The experimental groups included patients of similar age with clinically established diagnosis of POAG on the basis of complex examination including tonometry (ICare PRO tonometer, ICare, Finland), visometry, biomicroscopy, ophthalmoscopy, perimetry (Heidelberg Edge Perimeter, Heidelberg Engineering, Germany) and optical coherence tomography (Spectralis OCT2, Heidelberg Engineering, Germany) with estimation of the true thickness of the neuroretinal rim. Additional examination included retinal tomography (Heidelberg Retina Tomograph 3, Heidelberg Engineering, Germany) with determination of the area and volume of the neuroretinal rim, the average thickness of the retinal nerve fiber layer at the disc margin, and the cap-to-disc ratio (C/D). As a result, patients were categorized as stages 2-4 of POAG. Stage 2 (moderate POAG) included individuals with changes in the paracentral visual field (narrowing of more than 10° in the nasal hemifield) and moderate excavation in at least one sector reaching the fundus edge. The mean C/D in these patients was ~0.6-0.7. Stage 3 (advanced glaucoma) included patients with a concentrically narrowed visual field (less than 15° from fixation in at least one segment) and subtotal marginal excavation of the optic nerve. The mean C/D value in patients with this stage was ~0.85. Patients with stage 4 (terminal glaucoma) were characterized by complete excavation of the optic nerve and significant vision loss. Most patients received hypotensive therapy including the use of prostaglandin analogs, beta-adrenoblockers, carboanhydrase (CA) inhibitors, and/or alpha-2-adrenomimetics for at least 1 year before surgery with IOP control once every 2 months. Demographic characteristics, comorbidities, and treatment regimens of study participants are summarized in Supplementary Data 1. Mean age (one-way ANOVA *p* = 0.168) and gender ratio (Fisher test *p*-value 0.2633) of patients showed no significant differences between the control group and groups with different glaucoma stages, nor between the control group and the total POAG group. There were no statistically significant differences in proportions of the most frequent self-reported comorbidities, such as arterial hypertension or coronary heart disease (Supplementary Data 2, Supplementary Fig. 7).

The studies involving human participants were conducted in accordance with the Declaration of Helsinki and the ARVO statement and were approved by the local ethical committee of the Helmholtz National Medical Research Center for Eye Diseases (No. 58, 17.03.2022). All participants signed written informed consent. All ethical regulations relevant to human research participants were followed.

### Collection and accumulation of AH from human patients

AH was collected during phacoemulsification followed by intraocular lens implantation in patients with cataract and nonpenetrating deep sclerectomy in patients with POAG. Sampling was performed by a qualified surgeons (S.Yu.P., O.M.F) under peribulbar (2% lidocaine, Pharmsynthez, Russia) and local (0.4% oxybuprocaine, Sentiss Pharma, India) anesthesia. Specifically, corneal paracentesis was performed using an ophthalmic knife (1.2 mm), 50 μL of AH was aspirated with a syringe, aliquoted into disposable tubes without special treatment, and stored at -80°C. Sample accumulation was performed according to the schedule of surgical interventions at the Helmholtz National Medical Research Center for Eye Diseases (5-7 samples per week).

### Determination of zinc content in human AH by atomic absorption spectrometry

Immediately before the measurements, AH samples were thawed, stirred, incubated at 25 ^o^C for 20 min, and diluted 20-80 times with a solution containing 0.5 mM Mg(NO_3_)_2_ (#42992, Fluka, USA) and 0.5 mM EDTA (#Am-O322, Amresco, USA), which were added to reduce the interference effect of chloride ions, the presence of which may affect accuracy of Zn^2+^ content measurements^94^. The dilution was carried out by mass such that the Zn^2+^ concentration falls in the range of the linear region of the calibration plot, but not less than twice the diluent signal. The recalculation of the mass coefficient to the volume coefficient was performed using the densities of AH (ρ = 1.017 g/ml) and the diluent (ρ = 0.9968 g/ml) determined in a separate series of experiments. Calibration was performed in the range of 0.5-8 μg/L Zn^2+^ using a standard solution of Zn(NO_3_)_2_ (#18827, Fluka, USA). To increase the reliability of measurements, the calibration solutions were supplied with 3 mM Cl^-^, which corresponds to the concentration of chloride ions in human AH^95^. The influence of chloride ions was found to be negligible, as the presence of this compound did not affect the calibration plots (Supplementary Fig. 8). Zinc content was measured using an atomic absorption spectrometer with electrothermal atomization and Zeeman background correction (iCE 3000, Thermo Fisher Scientific, USA) in an uncoated pyrographite cuvette at a wavelength of 213.8 nm. Argon of purity >99.999% was used to purge the cuvette. The optimized measurement program is given in Supplementary Data 12. Each AH sample was examined in a series of 3-6 parallel dilutions with the absorption signal recorded at least three times.

### Identification of zinc-binding metabolites in human AH by gas chromatography–mass spectrometry (GC-MS)

Thirty microliters of AH samples were placed in 2 mL tubes, centrifuged at 3000 × g at 4 °C for 5 min. A 20 µL of the supernatant obtained after centrifugation was mixed with internal standard and 70 µL of cold methanol/chloroform (Folch’s buffer) (v/v = 3/1). After incubating the mixture in a freezer at −20 °C for 20 min and centrifugation at 14,000 × g and 4 °C for 20 min, the supernatant was carefully transferred to an autosampler tube. All samples in autosampler vials were briefly evaporated to remove chloroform using a CentriVap vacuum concentrator (Labconco, USA). For derivatization step 1, 25 μL of methoxylamine hydrochloride (anhydrous) in pyridine (20 mg mL^−1^) was added to the dry sample and incubated under shaking (1350 rpm) at 37 °C for 1.5 h. The sample was evaporated to dryness under a light stream of nitrogen at 21 °C^96^ and trimethylsilylated with 50 μL of N,O-Bis(trimethylsilyl)trifluoroacetamide for 30 min, 1350 rpm, 37 °C. The obtained samples were analyzed on a GCMS-QP2020 gas chromatography-mass spectrometer (Shimadzu, Japan). A one-microliter aliquot was injected in splitless mode using an AOC-20i autosampler injector into a deactivated split liner (#221-48335-01, Shimadzu, Japan), and the sampling time was 1 minute. The separation was performed on a 30 mm × 0.250 mm × 0.5 μm HP5-MS column (#19091S-433, Agilent, USA). The injector temperature was 280 °C, the carrier gas was helium, and the column flow rate was 1.1 mL/min. The oven temperature was set at 100 °C for 4 min, then heated at a rate of 10 °C/min to 320°C and held for 11 min at 320 °C. The ion source temperature was 200 °C, the interface temperature was 280 °C, the solvent extraction time was 4 min, the detector voltage was 1.0 kV, and the mass determination range was 45 to 600 Da. Metabolites were assigned using the Shimadzu Smart metabolite database (#225-28366, Shimadzu, Japan). Metabolite data were analyzed using MetaboAnalyst 5.0 software (www.metaboanalyst.ca)^97^. Nonadecanoic acid, ethyl ester was used as an internal standard. The significance of each metabolite (control versus each of the POAG groups) was assessed by Student’s t-test with adjusted false discovery rate (FDR). To select metabolites significantly contributing to the prediction of different classes, the PLS-DA model was generated on mean-centered and unit variance scaled data. Differential compounds were selected by assessing the importance of the variable in prediction (VIP), using criteria of VIP > 1 and false discovery rate (FDR)-adjusted *p*-value < 0.05. The Pheatmap (v1.0.12) R package was applied to construct a heatmap on autoscaled data using Euclidean distances for row clustering. Classification of the identified compounds was performed using the ClassyFire program^98^. Quantitative and qualitative analysis of the zinc-chelating features of metabolites showing significant changes in POAG was performed using the IUPAC stability constants (SC) database^99^, NIST46 database^100^ and DrugBank database^101^.

### Analysis of the antioxidant activity of human AH

The total antioxidant activity of AH was analyzed using the hemoglobin/H_2_O_2_/luminol system as described in our previous work^102^. The method is based on the inhibition of chemiluminescence of oxidized luminol by scavenging ROS (formed by the interaction of hemoglobin with hydrogen peroxide) by low molecular weight antioxidants present in AH. Briefly, AH samples were diluted 1:200 in PBS, mixed with 0.01 mM luminol and 0.5 mM hemoglobin, and the reaction was induced by adding H_2_O_2_ to a final concentration of 6 μM. Chemiluminescence was recorded every 1 s for 10 min. Calibration was performed using 1-8 μM Trolox (6-hydroxy-2,5,7,8-tetramethylchroman-2-carboxylic acid) standard solutions, and results were presented in Trolox equivalent. Hemoglobin (#H2625), luminol (#123072), hydrogen peroxide (#216763), and Trolox (#238813) were from Sigma-Aldrich (USA). Glutathione peroxidase (GPx) and superoxide dismutase (SOD) activities in AH were determined colorimetrically using Ransel and Ransod kits (#RS504 and SD125, Randox, UK). All reactions were monitored using a CLARIOstar Plus Reader (BMG Labtech, Germany).

### Rabbit model of ocular hypertension: clinics, electrophysiology and morphology

The study involved 26 six-month-old New Zealand white male rabbits weighing 2.3-3 kg (Manikhino, Russia) without ocular pathologies, which were kept in individual cages under a 12-hour light/dark cycle at 22–25 °C and 55–60% humidity with free access to feed and water. This species was chosen based on greater similarity to humans in terms of biochemical and biomechanical properties of the visual system, compared to rodents^103^. All experiments were performed under general anesthesia induced by intramuscular injection of 1:2 mixture of 50 mg/mL tiletamine/zolazepam (Zoletil 100, Virbac, France) and 20 mg/mL xylazine hydrochloride (Nita-Farm, Russia). Animals were additionally treated with the local anesthetic Alcaine (Alcon-Couvreur N.V., S.A., Belgium) before intraocular injections. In most of cases, a model of ocular hypertension (methylcellulose occlusion model) was induced in the right eye of the animals according to a previously described procedure^17^, and the left eye was used as a healthy (intact) control. Briefly, 150 μL of AH was aspirated from the anterior chamber with a 30-gauge needle and an equal volume of 2% methylcellulose was injected using the same needle and a different syringe. The development of glaucoma neuropathy was monitored in 16 animals using ophthalmotonometry, histology, and immunohistochemistry, as well as scotopic electroretinography (ERG) to record the activity of the outer retina^20,104^. Rabbits with the characteristics described above were randomly divided into two groups of 8 animals each without establishing additional criteria or confounder control. Information on group composition was available only to the veterinarian (VVT). Group size and composition were determined by the ethics committee to minimize the number of animals while ensuring the accuracy of the results. IOP was measured using a TonoVet tonometer (Icare, Finland) every 1 h for the first 7 h and once a day from the second to the seventh (group 1) or fourteenth (group 2) day after injection. In the animals of group 2, before and on the fourteenth day after injection, ERGs were recorded using the RETIport ERG visual electrophysiology system (An-vision, Germany) as described in ref. ^102^. For histological analysis of the posterior sector of the eye^102,105^, animals from both groups were euthanized by an overdose of anesthesia, eyes were enucleated and fixed in 10% formalin on phosphate buffer (pH 7.4) for 24 h. The samples were dehydrated, embedded in paraffin, and 3 μm thick slices were made on a Leica RM 2245 rotary microtome (Leica, Germany). Paraffin sections were mounted on slides, dried, deparaffinized, hydrated, stained with Carazzi’s hematoxylin and water-alcohol eosin, and placed under coverslips in BioMount medium (Bio-Optica, Italy). Unless otherwise stated, reagents for histologic examination were from Biovitrum (Moscow, Russia). The obtained histological preparations were analyzed on a Zeiss Axio Observer A1 light microscope (Carl Zeiss, Germany) equipped with a high-resolution color digital microscopy camera AxioCam 305 (Carl Zeiss, Germany) using Zeiss Zen 2 lite blue edition software (Carl Zeiss, Germany). Microphotographs were processed using AxioVision v.3.0 software (Carl Zeiss, Germany). Morphometric analysis of the count and state of RGCs was performed using the Aperio ImageScope software (Leica, Germany). The length of a retinal section in μm was measured as the sum of sections using a ruler. The total number of RGCs and the number of pycnotic (apoptotic) cells were counted in the selected section using a counter at high magnification (400×). For each eye, 2-4 retinal sections were quantitatively examined so that the size of the examined area was at least 6800 μm. In all animals, analysis was performed for sections obtained at an equal distance of 5 mm from the visual streak along the nasotemporal axis. For immunocytochemical staining, the formalin-fixed eyes with removed cornea and lens were incubated in 10% and 30% sucrose solutions for 6 h and 24 h, respectively, and transferred to cryotomy medium (OCT; Sakura, Kobe, Japan) followed by rapid freezing. 40 μm thick cross-cryosections were placed on Superfrost Plus slides (Fisher Scientific, Loughborough, UK). For ICC analysis, the samples were thawed, fixed in buffered formalin (20 min at room temperature), and subjected to antigen release procedure using Tris-EDTA solution (pH 9.0) by heating in a steamer (30 min). After washing with PBS, the sections were incubated with blocking buffer (PBS, containing 0.1% Tween, 0.01% NaN_3_ and 10% goat serum) at room temperature and incubated with primary antibody against Brna3a (1:500, clone 5A3.2, Sigma-Aldrich, USA) for 24 h at 4°C. After washing 5 times in PBS, the sections were incubated in a solution of secondary rabbit antibody against mouse IgG (H + L) conjugated to Alexa Fluor® 546 (1:1000, Thermo Fisher Scientific, USA) in blocking buffer. Images were acquired on an Eclipse Ti-E microscope with an A1 confocal module (Nikon Corporation, Japan) and a Plan Fluor 40 × 1.3 objective and analyzed using NIS-Elements and ImageJ programs.

### Rabbit model of ocular hypertension: collection of AH and retina samples and determination of zinc content and antioxidant activity

The model of ocular hypertension was induced as described above in a group of 10 rabbits (group 3). After induction, the animals were maintained for 7 days with daily IOP monitoring. In all specimens under general anesthesia on the 7th day after methylcellulose injection, AH was aspirated from the anterior chamber of control and experimental eyes, aliquoted, and stored at −80 °C until further studies. Zinc content, AOA and antioxidant enzymes (GPx and SOD) activities were determined exactly as described for human AH samples. To determine retinal zinc content, some of the animals were euthanized on day 7 after methylcellulose administration by anesthetic overdose. Enucleation eyeballs was performed postmortem, and retinas were isolated surgically immediately after scarification and stored at −80 °C^105^. After thawing, 40 mg of retinal sample was homogenized with a Teflon pestle, dried to constant mass at 0.6 Pa for 75 h, and the mass of dry residue per 1 g of retina was estimated. Separately, 20 mg of the retinal sample was incubated with 170 μL of 30% H_2_O_2_ (#16911, Sigma-Aldrich, USA) and 10 μL of 0.1 M HNO_3_ overnight at 65 °C, centrifuged for 5 minutes at 10000 rpm, and diluted 100–300 times with aqueous solution of 0.5 mM Mg(NO_3_)_2_ (#16911, Sigma-Aldrich, USA) and 0.5 mM EDTA (#Am-O322, Amresco, USA). Zinc content in the obtained samples was determined by AAS as described for AH samples, using standard solutions of Zn(NO_3_)_2_ in the concentration range of 0.5-8 µg/L to construct a calibration curve.

### Ethics statement

Animal studies were performed according to the 8th edition “Guide for the Care and Use of Laboratory Animals” of the National Research Council and “Statement for the Use of Animals in Ophthalmic and Visual Research” of the Association for Research in Vision and Ophthalmology (ARVO). The protocol was approved by the Bioethics Commission of the Belozersky Institute of Physico-chemical Biology and the Faculty of Bioengineering and Bioinformatics of Lomonosov Moscow State University (No. 005-5/9/2023, 30.06.2023). We have complied with all relevant ethical regulations for animal use.

### Prediction of zinc-binding sites in core proteins of human AH proteome

The primary screening for the presence of Zn^2+^-binding sites in AH proteins was performed in silico. All proteins from the constitutive AH proteome described in ref. ^25,26^ excluding immunoglobulins (a total of 38 proteins) were analyzed (Supplementary Data 10). Three-dimensional structures of candidate proteins were obtained from the RCSB Protein Data Bank (https://www.rcsb.org/). The structures obtained in the absence of ligands and corresponding to the major conformers of each protein were selected (a total of 26 proteins, Supplementary Data 10). In the case where multiple structures were resolved for a single protein, the structure with the highest resolution was prioritized. Potential areas of zinc coordination in the molecules of the selected proteins were searched using the machine learning-based ZincBindPredict program (https://zincbind.net), which uses classifiers trained on the ZincBind database containing zinc-binding site structures automatically generated from the RCSB Protein Data Bank^27^. Potential zinc coordinators were defined as sulfur, nitrogen, or oxygen atoms of the major Zn^2+^-chelating residues (C, D, E, and H) localized within 3 angstroms of the zinc atom. The results of the extended prediction of Zn^2+^-binding sites in existing PDB and AlphaFold (https://alphafold.ebi.ac.uk) structures of AH proteins^25,26,106^ (a total of 155 proteins) are summarized in Supplementary Data 11.

### Cloning, expression, and purification of PEDF

The genetic construct for expression of untagged human PEDF lacking signal peptide (Supplementary Fig. 9a) in bacterial cells was obtained using standard techniques^107^. Total RNA was isolated using the ExtractRNA kit (#BC032, Evrogen, Russia) from human retinoblastoma Y79 cells (4.5 × 10^6^ cells) cultivated as described below. Complementary DNA was synthesized on PEDF mRNA template with MINT reverse transcriptase (#SK003, Evrogen, Russia) using a primer (5‘-ATTGGAATATTAAACTGG-3‘) complementary to the 3’-untranslated region. The obtained cDNA was amplified by PCR using a forward (5‘-ATACATATGCAGAACCCTGCCAGCCCCC-3’) and reverse (5’-ATAGTCGACTTAGTAGGGGCCCCTGGGGGGGTCC-3‘) primers. The PCR product was cloned into the expression vector pET-22b(+) (Novagen, USA) between the *NdeI* and *SalI*. The obtained plasmid DNA was analyzed by sequencing on an ABI PRISM 310 genetic analyzer (Applied Biosystems, USA).

PEDF expression was performed in *E. coli* BL21(DE3)Star strain cultured in 2YT medium (#210929, Difco, USA) containing 100 μg/mL ampicillin and 0.04% glucose. Cells transformed with plasmid encoding PEDF were cultured at 37 °C up to OD_595_ = 1, incubated overnight at 4 °C, diluted 1:80 with the same medium, cultured up to OD_595_ = 0.6, and PEDF expression was induced in the presence of 1 mM IPTG (#AFF-J17886-14, Thermo Fisher Scientific, USA) for 4 h at 37 °C. The cells were collected by centrifugation (3 000×g), resuspended in lysis buffer (50 mM Tris-HCl (#A1379, AppliChem, Germany) pH 8.0, 0.5 mM EDTA (#Q2325, Thermo Fisher Scientific, USA), 5 mM MgCl_2_ (#141396, AppliChem, Germany), 3 mM DTT (#A2948, AppliChem, Germany), 5% glycerol (#G5516, Merck, USA), 50 μg/mL lysozyme (#A4972, AppliChem, Germany), and frozen at -20°C. After thawing, the suspension was mixed with 2 mM PMSF (#A0999, AppliChem, Germany), and 0.2% NP-40 (#492016, Millipore, USA), stirred for 10 min, sonicated on ice for 5 min, and the soluble proteins were separated from the cellular debris by centrifugation (17,000 × g). The supernatant was loaded onto DEAE-Sepharose (#17070910, Cytiva, USA) equilibrated with buffer A (20 mM Tris-HCl, pH 7.5), the column was washed with buffer A containing 100 mM NaCl (#A2942, AppliChem, Germany), and the PEDF fractions were eluted with a linear gradient from 100 to 300 mM NaCl in the same buffer (Supplementary Fig. 9b). The eluate was dialyzed against buffer A (for 8 h at 4 °C), loaded onto heparin-Sepharose (#17099801, Cytiva, USA) equilibrated with buffer A, the column was washed with buffer A containing 100 mM NaCl, and the PEDF fraction was eluted with the same buffer containing 150 mM NaCl. After dialysis against buffer A (4°C, overnight), the fraction was loaded onto MonoQ (#17516601, Cytiva, USA) equilibrated with buffer A, the column was washed with buffer A containing 100 mM NaCl, and PEDF was eluted by a linear gradient from 100 to 300 mM NaCl in the same buffer. Contaminating divalent ions were removed from the resulting protein preparation by treatment with a 10-fold excess of EDTA and removal of chelate complexes by gel filtration on Sephadex G-25 (#17003302, Cytiva, USA). The molecular weight and homogeneity of the obtained recombinant PEDF were confirmed by high-performance liquid chromatography-mass spectrometry (Shimadzu LCMS-2010EV single quadrupole mass spectrometer (Shimadzu Co., Kyoto, Japan), C18 reversed-phase column (Phenomenex, USA)) and SDS-PAGE, and its purity exceeded 95% (Supplementary Fig. 9b–d). The CD spectra of the obtained form of PEDF (for the method details, see below) were in line with those expected for a globular protein and consistent with previously determined spectra of human and mouse PEDF^108,109^, indicating proper folding of the recombinant protein (Supplementary Fig. 9e). Recombinant PEDF demonstrated the same neurotrophic activity in cellular models (see Fig. 5), as reported in previous studies^34,36,110^.

### Verification of zinc-binding properties of human AH proteins by differential scanning calorimetry (DSC)

DSC thermograms of human recombinant label/tag-free cystatin C (#8CY5, Hytest, Russia), VDBP (#G8764, Sigma-Aldrich, USA), and PEDF were registered using a differential scanning microcalorimeter MicroCal PEAQ-DSC (Malvern Panalytical, UK) as described in ref. ^111^. The measurements were performed in 130 μL of sample containing 50 μM protein without zinc or with the addition of a 2-fold (“low”) or 10-fold (“high”) molar excess of ZnCl_2_ (#108816, Merck, Germany). The thermograms in the temperature region from 25 to 100 °C were recorded at a heating rate of 1 °C/min. Thermal denaturation (melting) temperatures (T_m_) were calculated using the pre-installed MicroCal PEAQ-DSC software (Malvern Panalytical, UK) according to a “non-two-stage” fitting model.

### Determination of affinity and stoichiometry of zinc binding to PEDF by equilibrium dialysis coupled with AAS

Zinc binding to PEDF was studied using a 96-well micro-equilibrium dialysis system (HTDialysis LLC, USA) as reported in ref. ^112^. One half of each well was filled with 100 μL of (4.6–20) μM of PEDF solution in 20 mM Hepes-KOH buffer (#54457, Sigma-Aldrich, USA), pH 7.3, and the other half contained 100 μL of the same buffer with (2–200) μM ZnCl_2_ (#108816, Merck, Germany) without PEDF. The solutions in the wells were equilibrated by continuous shaking (130 rpm) at 25 °C for 15–17 h. The total concentrations of zinc in equilibrated solutions were measured by AAS as described above. The concentration of Zn^2+^ bound to PEDF was estimated for each well as the difference between total metal concentrations measured for both halves of the well, assuming that the concentration of free zinc in two halves of the well was the same.

### Study of the effect of zinc binding on PEDF surface hydrophobicity by bis-ANS fluorescence method

Bis-ANS fluorescence studies were carried out at 20°C on a Cary Eclipse spectrofluorimeter (Varian, Inc., USA), equipped with a Peltier-controlled cell holder as described in ref. ^113^. Quartz cells with a pathlength of 10 mm were used. The concentrations of PEDF and bis-ANS (#D4162, Sigma-Aldrich, USA) were 2.7 μM and 0.5 μM, respectively. Bis-ANS fluorescence was measured in 20 mM HEPES-KOH, pH 7.3, with excitation at 385 nm. The maximum intensities of fluorescence emission spectra were obtained by fitting the spectra to lognormal function using the LogNormal program (IBI RAS, Pushchino). The affinity of PEDF for zinc at 20 °C was estimated by spectrofluorimetric titration of the protein in the presence of bis-ANS with standard ZnCl_2_ solutions.

### Study of the effect of zinc binding on PEDF secondary structure by circular dichroism (CD) spectroscopy

Far-UV CD spectra of PEDF (2.7 µM) were measured at 37 °C with J-810 spectropolarimeter (JASCO, Inc., Japan), equipped with a Peltier-controlled cell holder. The instrument was calibrated with an aqueous solution of d-10-camphorsulfonic acid according to the manufacturer’s instruction. The cell compartment was purged with N_2_. Cell with pathlengths of 1.00 mm was used. Measurements were performed in Hepes-KOH, 100 mM KCl, pH 7.6 buffer, either under metal-free conditions (1 mM EDTA) or in the presence of a 2-fold or 10-fold excess of ZnCl_2_. Bandwidth was 2 nm, averaging time 2 s, accumulation 3.

### Study of zinc-dependent properties of PEDF by differential scanning fluorimetry (nanoDSF)

Label-free fluorimetric analysis of Zn^2+^-dependent thermally induced structural changes in PEDF was performed according to the previously described methodology^114^. Briefly, intrinsic tryptophan fluorescence and light scattering of PEDF (10 µM) were monitored in 20 mM Tris-HCl, pH 7.5 in the presence of 5–100 μM Zn^2+^ over the temperature range of 25–95 °C using Prometheus Panta instrument (NanoTemper Technologies, Munich, Germany). Mid-transition melting temperatures (T_m_) for each sample were calculated based on the temperature dependence of the first derivative of tryptophan fluorescence ratio (F_350_/F_330_). The nanoDSF data were analyzed using PR.Panta Analysis software (NanoTemper Technologies, Munich, Germany).

### Study of the effect of zinc binding on PEDF oligomeric state by dynamic light scattering (DLS)

DLS measurements of PEDF at high zinc levels were performed on a Zetasizer Advance instrument (Malvern Panalytical, UK) using the previously described settings^114^. Samples containing 25 μM PEDF in the presence of 25–500 μM ZnCl_2_ were placed in 70 μL Ultra-Micro UV cuvettes (#759215, BrandTech, USA), and five repeated measurements were performed at 37 °C. Zinc-dependent oligomerization/aggregation of PEDF was assessed by measuring intensity weighted mean hydrodynamic size (Z-average) and polydispersity index (PDI) of the particles in the sample. To visualize the observed polymerization of PEDF at high excess Zn^2+^, which was partially abolished by the addition of 5 mM EGTA, a graph of particle size distribution by volume was plotted. Data analysis was performed using the ZS Explorer software version 3.2.1 supplied by the manufacturer (Malvern Panalytical, UK). DLS measurements of PEDF at low zinc levels were performed with a Prometheus Panta instrument (NanoTemper Technologies, Munich, Germany) under the same conditions using 10 μM protein and 0–50 μM ZnCl_2_. The DLS data were analyzed using PR.Panta Analysis software (NanoTemper Technologies, Munich, Germany).

### PEDF crystallization, data collection, structure solution, and refinement

An initial crystallization screening of PEDF was performed with a robotic crystallization system (NT8, Formulatrix, USA) and commercially available 96-well crystallization screen JCSG-plus (Molecular Dimensions, USA) and PACT Premier (Molecular Dimensions, USA) at 20 °C using the sitting drop vapor diffusion method. The protein concentration was 11 mg/mL in the following buffer: 20 mM Tris-HCl, 75 mM NaCl, 5 mM ZnCl_2_, pH 8.2. The protein buffer was supplemented with 5 mM ZnCl_2_ to ensure saturation of zinc-binding sites during crystallization. The crystals were obtained within a week under the following conditions: 0.2 M Magnesium chloride hexahydrate, 0.1 M Tris-HCl pH 8.5, 20% w/v PEG 8000 (P2_1_2_1_2 space group), and 0.2 M Sodium sulfate, 20% w/v PEG 3350 (P2_1_2_1_2_1_ space group).

Crystals were wash-frozen in liquid nitrogen before data collection. The X-ray data from P2_1_2_1_2 crystal were collected at 100 K at beamline BL17U1 of the Shanghai Synchrotron Radiation Facility (SSRF, Shanghai, China). The X-ray data from P2_1_2_1_2_1_crystal were collected from at 100 K at beamline ID30B of the European Synchrotron Radiation Facility (ESRF, Grenoble, France) (Supplementary Data 13). The data were indexed, integrated, and scaled using the XDS program^115^. We determined the resolution cutoff based on the significance of the correlation between intensities from random half-datasets, as identified by XDS and recommended previously^116^. The structure was solved using the SIMBAD automatic pipeline (SIMBAD^117^, CCP4^118^, CCTBX^119^, AMORE^120^, MOLREP^121^, REFMAC^122^) with a PDB 1IMV^123^ as a model. The structure contained one and five molecules per asymmetric unit in space groups P2_1_2_1_2 and P2_1_2_1_2_1_, correspondingly (Supplementary Fig. 10). ModelCraft^124^ from CCP4 Cloud^125^ and phenix.refine^126^ from PHENIX^127^ were used for model rebuilding and refinement, respectively. The visual inspection of electron density maps and the manual model building were carried in COOT^128^.

Datasets were collected with X-ray wavelengths of 1.245 Å and 1.24 Å for the P2_1_2_1_2 and P2_1_2_1_2_1_ crystals, respectively, both above the Zn K-absorption edge. Zinc ions were positioned based on peaks (>7σ) on anomalous maps built in PHENIX (Supplementary Fig. 11). Anomalous difference maps were calculated in phenix.refine as a default procedure when both F(+) and F(−) (or I(+)/I(−)) are provided during refinement. Additional datasets were collected from similar crystals at 1.345 Å, which is well below the Zn K-absorption edge. Anomalous maps for these datasets showed no peaks, confirming the correctness of Zinc ion identification. X-ray fluorescence spectra were collected at ID30B (Supplementary Fig. 12) and confirmed the presence of Zinc ions in the samples.

Final models are deposited into the Protein Data Bank under accession codes 9J3Q (P2_1_2_1_2 space group) and 9J3P (P2_1_2_1_2_1_ space group); refinement statistics are shown in Supplementary Data 14. The electrostatic surface is calculated in APBS Electrostatics Plugin for PyMOL^129^.

### Monitoring of zinc stress and PEDF secretion in cellular models

The cytotoxic effects of zinc and PEDF secretion were studied on human retinoblastoma Y79 cells (#ACC-246, DSMZ-German Collection of Microorganisms and Cell Cultures GmbH, Germany) and human retinal pigment epithelium ARPE−19 cells (Cell Culture Collection of the Koltzov Institute of Developmental Biology of the Russian Academy of Sciences, Russia). Y79 cells were cultured in RPMI-1640 medium (Paneco, Russia), supplemented with 20% fetal bovine serum (FBS, Gibco, USA) and 4 mM L-glutamine (Paneco, Russia) at 37 °C in a humidified atmosphere with 5% CO_2_. For each experiment, cells were seeded into a 6-well (7.5 × 10^5^ cells per well) or 24-well plate (2.5 × 10^5^ cells per well) and cultured for 72 h. Prior to zinc treatment, cells were subjected to serum deprivation for 2 h, and sterile aqueous ZnSO_4_ (Sigma-Aldrich, USA) solution was added to each well directly into the media to a final concentration of 100 μM for 1, 2, 4, 6, or 8 h^130^. Y79 cell viability was assessed using the EZ4U Cell Proliferation & Cytotoxicity Assay kit (#BI-5000, Biomedica, Austria), which measures mitochondrial succinate dehydrogenase activity in living cells, and the Cytotoxicity Detection kit (#11644793001, Roche, Switzerland), based on measuring lactate dehydrogenase (LDH) leakage from damaged cells. The course of colorimetric reactions was monitored using a Synergy H4 multi-mode microplate reader (Biotek, USA). To monitor apoptosis, Y79 cells were washed with ice-cold DPBS and stained with the PE annexin V from the apoptosis detection kit (BD Biosciences, USA) according to the manufacturer’s instructions. The number of cells in early apoptosis was determined by the fluorescence intensity of PE annexin V on a MACSQuant Analyzer 10 flow cytometer (Miltenyi Biotec GmbH, Germany). To assess PEDF secretion, conditioned medium from Y79 cells was collected 1, 2, 4, 6, or 8 h after zinc addition.

ARPE−19 cells were cultured in PC-1 medium (Lonza, USA) supplemented with 10% FBS (HyClone, USA), 2% PC-1 Supplement (Lonza, USA), 2 mM L-glutamine (Paneco, Russia) and 5 μg/mL gentamicin (Gibco, USA) in a humidified atmosphere with 5% CO_2_. Before starting the experiments, ARPE−19 cells were weaned from FBS-containing media according to manufacturer’s protocol. Briefly, for seven weeks, the medium was replaced once a week with complete PC-1 medium with gradually decreasing serum concentration from 5%, 2.5%, 1.25%, 0.75%, 0.5%, 0.25% to 0%. Cells cultured in serum-free medium were seeded into 96-well plates (5000 cells per well) and cultured for another 3 days until 80% confluency. ZnSO_4_ diluted in serum-free PC-1 medium was added to the cells to a final concentration of 100 μM and cultured for 24 or 48 h. The viability of APRE19 cells was assessed by MTT (3-(4,5-dimethylthiazol-2-yl)-2,5-diphenyltetrazolium bromide) test. Briefly, 10 µl of MTT (5 mg/mL in PBS) was added to each well and the plate was incubated for 4 h. After incubation, the medium was removed, the formed formazan crystals were dissolved in 100 μl of DMSO, and the optical density of the resulting solutions was analyzed at 550 nm using Synergy H4 multi-mode microplate reader (Biotek, USA). Alternatively, APRE19 cell viability was analyzed microscopically by double staining of live/dead cells with fluorescein diacetate (FDA) and propidium iodide (PI). The staining solution, containing 5 ml DMEM, 8 μl FDA (5 mg/ml) and 50 μl PI (2 mg/ml) was added to the wells, cells were incubated at room temperature for 4-5 minutes in the dark, washed with PBS and examined on Eclipse Ti-E confocal laser scanning microscope (CLSM) with A1 confocal module (Nikon Corporation, Japan) and CFI Plan Apo VC 20×/0.75 objective lens with a life support system, maintaining normal culturing conditions (37°C, 5% CO_2_). Images were analyzed using NIS-Elements (Nikon Corporation, Japan) and ImageJ^131^ software. To assess PEDF secretion, conditioned ARPE−19 cells medium was collected 24 or 48 h after zinc addition.

### Evaluation of PEDF secretion by Western blotting

The content of secreted PEDF was analyzed in AH samples obtained in previous steps from POAG patients and animals with an ocular hypertension model, as well as in samples of conditioned medium of Y79 and ARPE−19 cells under zinc stress conditions. The loading of samples was normalized to the volume of AH or cellular medium. Specifically, 5 μL of human AH, 10 μL of rabbit AH, or 45 μL of the medium were mixed with 1/3 volume of 4x Laemmli Sample Buffer (#1610747, Biorad, USA) and subjected to 12% sodium dodecyl sulfate polyacrylamide gel electrophoresis (SDS-PAGE). Proteins were transferred to a nitrocellulose membrane using the Trans-Blot Turbo Transfer System (Biorad, USA). The membrane was blocked with 5% delipidated milk in PBS containing 0.1% Tween 20 (PBST) for 1 h, incubated with polyclonal anti-PEDF antibodies (#PAB972Hu01, Cloud-Clone corp., China, 1:1000 in PBST) for 1 h, washed with PBST, and incubated with horseradish peroxidase-conjugated anti-rabbit IgG antibody (#111-035-003, Jackson ImmunoResearch, UK, 1:50,000 in PBST) for 1 h. The PEDF band was visualized employing Clarity Western Enhanced Chemiluminescence (ECL) Substrate and ChemiDoc™ XRS+ gel documentation system (Bio-Rad, USA). For quantification, the amounts of PEDF were calculated by densitometric analysis of the bands using GelAnalyzer.2010a software (http://www.gelanalyzer.com/).

### Determination of the effect of zinc on PEDF axogenic activity in SH-SY5Y cells

The axogenic activity of PEDF was analyzed on the human neuroblastoma cell line SH-SY5Y (#CRL-2266, American Type Culture Collection, USA) differentiated as described in ref. ^132^. Cells were cultured in 1:1 EMEM/F12 medium (Paneco, Russia) supplemented with 15% heat-inactivated FBS (HyClone, USA), 2 mM L-glutamine (Paneco, Russia) and 100 μg/mL gentamicin (Gibco, USA) (phase 0 medium) at 37°C in humidified atmosphere of 5% CO_2_ with medium change every 3–4 days. Cell differentiation was performed in three phases using phase 1 medium (EMEM, 2.5% hiFBS, 2 mM L-glutamine, 100 μg/mL gentamicin), phase 2 medium (EMEM, 1% hiFBS, 2 mM L-glutamine, 100 μg/mL gentamicin), and phase 3 medium (Neurobasal medium (Invitrogen, USA), 2 mM L-glutamine, 2% NeuroMax 50× (Paneco, Russia), 100 μg/mL gentamicin, 20 mM KCl, 50 ng/ml NGF, 2 mM dibutyryl cyclic AMP). SH-SY5Y cells were seeded onto 6-cm dishes and cultured for 24 h in phase 0 medium, which was then replaced with phase 1 medium containing 10 μM all-trans-retinoic acid, and the cells were cultured with medium changes on days 3 and 5. On day 7, cells were plated at a 1:1 ratio, and after 24 h the phase 1 medium was replaced with phase 2 medium containing 10 μM trans-retinoic acid added immediately before the replacement. On day 10, cells were seeded on poly-L-lysine-coated glasses into 35 mm dishes (100,000 cells per dish), and after 24 h the medium was replaced with phase 3 medium. The effect of PEDF/zinc was investigated on day 13 of differentiation. For this purpose, dishes were randomly divided into two groups. Cells of the first group were cultured for 48 h in medium supplemented with an aliquot of 50 μM PEDF at a ratio of 1:10 (*v/v*; the final concentration of PEDF was 5 μM). Cells of the second group were cultured for the same time in medium supplemented with an aliquot of 50 μM PEDF preincubated with 100 μM zinc at 37 °C for 30 min, also at a ratio of 1:10 (*v/v*; the final concentration of PEDF was 5 μM). Cells were then fixed with 4% paraformaldehyde, treated with 0.1% Triton X-100 in PBS for 10 min, incubated with 0.1% Tween 20, 0.3 M glycine, and 1% BSA in PBS for 2 h and incubated overnight at +4 °C with monoclonal antibodies against β-III tubulin conjugated to Alexa Fluor® 647 (1:500, 657405, Biolegend, USA) in the same buffer. Cell nuclei were visualized using Hoechst 33342. Images were acquired using an Eclipse Ti-E confocal laser scanning microscope (CLSM) with an A1 confocal module (Nikon Corporation, Japan) and a Plan Fluor 40 × 1,3 objective. Images were analyzed using NIS-Elements (Nikon Corporation, Japan), ImageJ^131^ software and the NeuronJ plugin.

### Determination of the effect of zinc on differentiative activity of PEDF in Y79 cells

Neuronal differentiation of Y79 cells was performed by growing cells on poly-L-lysine/collagen-coated culture dishes in the presence of PEDF/zinc in conditioned medium obtained from differentiated SH-SY5Y cells (see above)^133^. The medium was filtered through a 0.22 µm membrane, and 5 μM PEDF or 5 μM PEDF and 1 μM ZnSO_4_ was added. Cells were cultured for 10 days with a double change of the medium. Cell fixation and visualization were performed exactly as described above for SH-SY5Y cells. The presence of neuronal outgrowths (diameter of one cell body) was used as a differentiation criterion.

### Evaluation of the neuroprotective activity of PEDF in Y79 cells

Zinc stress of Y79 cells was induced by adding 100 μM ZnSO_4_ to FBS-free medium and culturing cells for 2, 4, 6, or 8 h as described above, except that 15 min before zinc treatment, purified recombinant PEDF in 20 mM Tris-HCl, pH 8.0, was added to the medium to a final concentration of 1 μM. In control experiments, Y79 cells similarly pretreated with PEDF were subjected to oxidative stress induced by incubation with 300 μM H_2_O_2_ for the same periods of time. In both cases, cell viability was assessed using an LDH-based Cytotoxicity Detection kit (#11644793001, Roche, Switzerland).

### Determination of the effect of zinc on PEDF activity against PEDF-R in vitro

The effect of zinc on PEDF activity was determined in an assay involving native PEDF-R in ARPE−19 cell membranes according to the previously described methodology^134^, except that the phospholipase activity of the receptor was monitored by quantification of fatty acid (linoleic acid, LA) release from the substrate ([1,2-dilinoleoyl]-phosphatidylcholine) by HPLC-MS/MS. Specifically, ARPE−19 cells (150 cm^2^) were cultured as described above until 80% confluency, harvested, washed with PBS, and resuspended in 7 mL of homogenization buffer (20 mM HEPES, pH 7, 0.1 M KCl, protease inhibitors). After sonication (2 pulses of 5 s at 4 °C), the cell debris was separated by centrifugation at 1000 × *g* for 10 min, and the supernatant was ultracentrifuged at 80,000 × *g* for 30 min at 4 °C. The pellet was resuspended in 150 μL of 50 mM Tris-HCl buffer (pH 7.5), 100 mM NaCl, 0.1% NP-40 and the resulting PEDF-R-containing preparation (50 μL) was incubated with 5 μM PEDF and L-α-phosphatidylcholine (# P5638, Sigma-Aldrich, USA) in 50 mM Tris-HCl (pH 7.5), 3 mM deoxycholate with or without 50 μM ZnCl_2_ in a final volume of 0.5 mL at 37 °C for 20 min. The reaction was stopped by adding ice-cold methanol (1:2 v/v), the mixture was centrifuged 10,000 × *g* for 10 min to remove proteins, the supernatant was mixed with 0.1% acetic acid and loaded onto a solid-phase lipid extraction cartridge (Oasis ® PRIME HLB cartridge (60 mg, 3 cc)). Solid-phase extraction was performed using a cartridge manifold VacElut (Agilent, Santa Clara, CA, USA) with a laboport mini-pump (KNF, Hamburg, Germany). The cartridge was washed with 15% methanol containing 0.1% formic acid, and lipids were sequentially eluted with 500 μL of anhydrous methanol and 500 μL of acetonitrile. After the extraction, the samples were concentrated by evaporation of the solvent under a gentle stream of nitrogen, reconstituted in 100 μL of 90% methanol, and analyzed by an 8040 series UPLC-MS/MS mass spectrometer (Shimadzu, Tokyo, Japan). The lipid compounds were separated by reverse-phase UPLC (injection volume 20 μL) using Phenomenex C8 column (Kinetex® 2.6 µm C8 100 Å, LC Column 150 mm × 2.1 mm) with a flow rate of 0.4 mL/min and temperatures of the sample cooler and the column of 5 °C and 40 °C, respectively. The elution was performed using an acetonitrile gradient in 0.1% (v/v) formic acid. The changes in concentrations of acetonitrile were as follows: 10% (0 min)–25% (5 min)–35% (10 min)–75% (20 min)–95% (20.1 min)–95% (25 min)–10% (25.1 min)–0% (30 min). The LA was identified and quantified by comparing its negative ESI-MS spectra and retention times obtained for LA (#62230, Sigma-Aldrich, USA) and LA-d4 (#390150, Cayman Chemical, USA) standard samples ([M-H]-, m/z, peak area, m/z 279.2 and m/z 283.2, respectively).

### Analytical methods

All solutions were prepared using ultrapure water (>18.2 mΩ). Protein concentration was measured using Pierce bicinchoninic acid (BCA) Protein Assay Kit (#23225, Thermo Fisher Scientific, USA) or Bio-Rad protein assay dye (#5000006, Bio-Rad, USA), or according to ref. ^135^.

### Statistics and reproducibility

Demographic and medical characteristics between patient groups were compared using one-way ANOVA in the case of numerical parameters (age) or Fisher’s exact test in the case of categorical variables (gender and comorbidities). The effect of treatment regimens on zinc and metabolite content in AH was evaluated by Student’s *t*-test with FDR correction. The remaining experimental data were analyzed by the mean standard error method using SigmaPlot 11 software (Systat Software, Germany) for calculation and visualization. Plots and histograms in the figures represent the mean ± SEM. Statistical significance was assessed by Student’s t-test or Mann-Whitney U-test for pairwise comparisons. In all cases, a *p*-value of less than 0.05 was considered significant. At least 3 biological replicates were used in each experiment. The number of biological replicates (*n*) is detailed in the figure legends.

### Reporting summary

Further information on research design is available in the Nature Portfolio Reporting Summary linked to this article.

## Supplementary information

Supplementary Information

Description of Additional Supplementary Files

Supplementary Data 1-15

Reporting Summary

## Data Availability

Crystal structures of complexes PEDF with zinc are deposited into the Protein Data Bank under accession codes 9J3Q (P21212 space group) and 9J3P (P212121 space group). The source data underlying the plots in the main figures are given in Supplementary Data 15. Uncropped images of the blots presented in the main article are shown in Supplementary Fig. 13. The mass spectral data files of AH metabolites/zinc chelators associated with POAG identified by GC-MS-based metabolomic analysis are deposited at the Center for Computational Mass Spectrometry (MassIVE MSV000093951^136^). The other datasets used and/or analyzed during the current study are available from the corresponding author on reasonable request.
