# Supplementary material for: A role of pigment epithelium-derived factor in zinc-mediated mechanism of neurodegeneration in glaucoma
Source: Commun Biol. 2025 Jul 1;8:965. doi: 10.1038/s42003-025-08370-8 (PMC12215840; doi:10.1038/s42003-025-08370-8)
Supplement: Supplementary file 1 — Supplementary Information [file 42003_2025_8370_MOESM1_ESM.pdf]

# Supplementary Material for manuscript

## Title:

A role of pigment epithelium-derived factor in zinc-mediated mechanism of neurodegeneration in glaucoma


## Author list:

Dmitry V. Chistyakov[1,2], Anatoliy S. Belousov[3,4], Marina P. Shevelyova[5], Elena N. Iomdina[6], Viktoriia E. Baksheeva[1,7], Natalia G. Shebardina[1], Anastasia M. Moysenovich[8], Timofey K. Bulgakov[8], Sergey Yu. Petrov[6], Mikhail L. Shishkin[1,9], Sanchay S. Tulush[1,9], Veronika V. Tiulina[1], Ekaterina I. Pogodina[10], Olga S. Gancharova[1], Olga M. Filippova[6], Alexey V. Baldin[1], Sergey V Goriainov[2], Arina I. Nikolskaya[11], Arthur O. Zalevsky[12], Andrei A. Deviatkin[3], Alisa A. Vologzhannikova[5], Neonila V. Gorokhovets[13], Ekaterina A. Litus[5], Sergey V. Komarov[14], François Devred[7], Marina G. Sergeeva[1], Alexey V. Mishin[3], Sergey S. Bukhdruker[3], Lijie Wu[15], Evandro A. Araujo[16], Andrey A. Zamyatnin Jr[1,9,11], Ivan I. Senin[1], Dmitry V. Zinchenko[10], Philipp O. Tsvetkov[7], Valentin I. Borshchevskiy[3,17], Sergei E. Permyakov[5], Evgeni Yu. Zernii[1]*

## Author information:

[1]Belozersky Institute of Physico-Chemical Biology, Lomonosov Moscow State University, Moscow 119992, Russia
[2]Peoples' Friendship University of Russia (RUDN University), Moscow 117198, Russia
[3]Moscow Institute of Physics and Technology (National Research University), Dolgoprudniy 141700, Russia
[4]Sao Carlos Institute of Physics, University of Sao Paulo, poloTErRA, 13563-120, Sao Carlos, São Paolo, Brazil
[5]Institute for Biological Instrumentation, Pushchino Scientific Center for Biological Research of the Russian Academy of Sciences, Pushchino 142290, Russia
[6]Helmholtz National Medical Research Center of Eye Diseases, Moscow 105062, Russia
[7]Institut Neurophysiopathol, INP, Faculté des Sciences Médicales et Paramédicales, Aix Marseille Univ, CNRS, 13005 Marseille, France
[8]Faculty of Biology, Lomonosov Moscow State University, Moscow 119234, Russia
[9]Department of Biological Chemistry, Sechenov First Moscow State Medical University, Moscow 119991, Russia
[10]Branch of Shemyakin and Ovchinnikov Institute of Bioorganic Chemistry, Russian Academy of Sciences, Pushchino 142290, Russia
[11]Faculty of Bioengineering and Bioinformatics, Lomonosov Moscow State University, Moscow 119234, Russia
[12]Shemyakin-Ovchinnikov Institute of Bioorganic Chemistry of the Russian Academy of Sciences, Moscow 117997, Russia
[13]Institute of Translational Medicine and Biotechnology, Sechenov First Moscow State Medical University, Moscow 119991, Russia
[14]Skryabin Moscow State Academy of Veterinary Medicine and Biotechnology, 109472 Moscow, Russia
[15]iHuman Institute, ShanghaiTech University, 201210 Shanghai, China
[16]Brazilian Synchrotron Light Laboratory (LNLS), Brazilian Center for Research in Energy and Materials, Campinas 13083-970, São Paulo, Brazil
[17]Joint Institute for Nuclear Research, Dubna 141980, Russia

*Correspondence to be addressed to Evgeni Yu. Zernii: zerni@belozersky.msu.ru

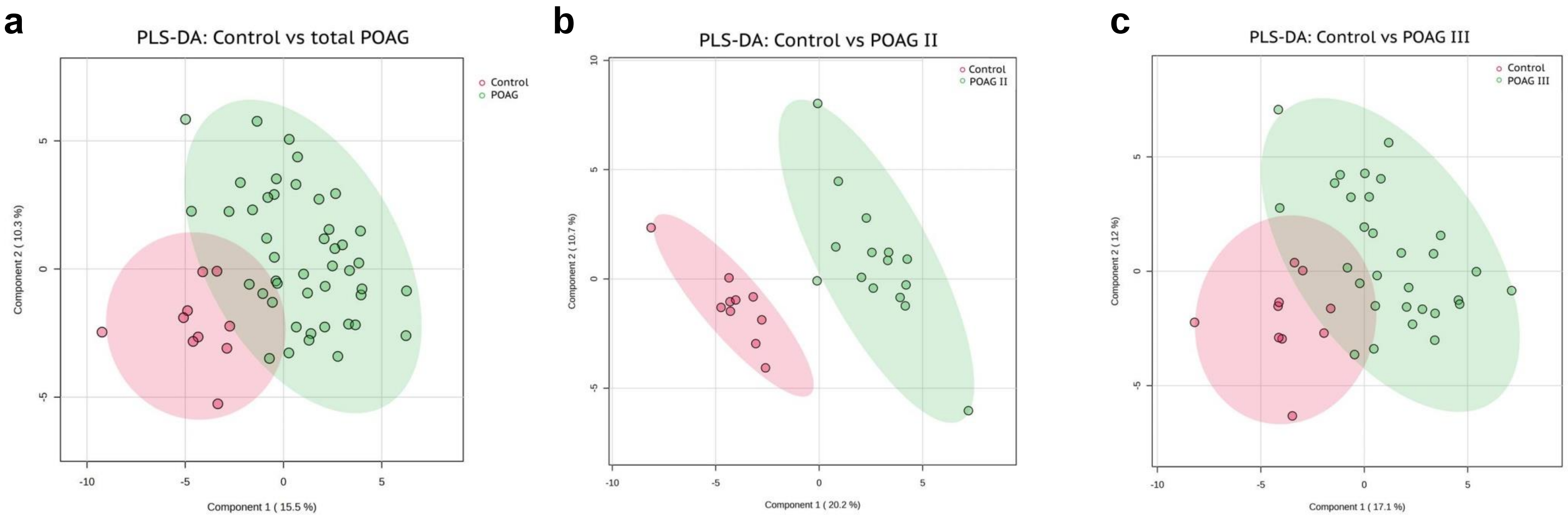


**Supplementary Figure 1**. The partial least squares discriminant analysis (PLS-DA) plot showing significant differences between the control group of patients (red) and (**a**) the total POAG group, (**b**) the stage 2 POAG group, and (**c**) the stage 3 POAG group (green) in the content of AH metabolites identified by GC-MS.

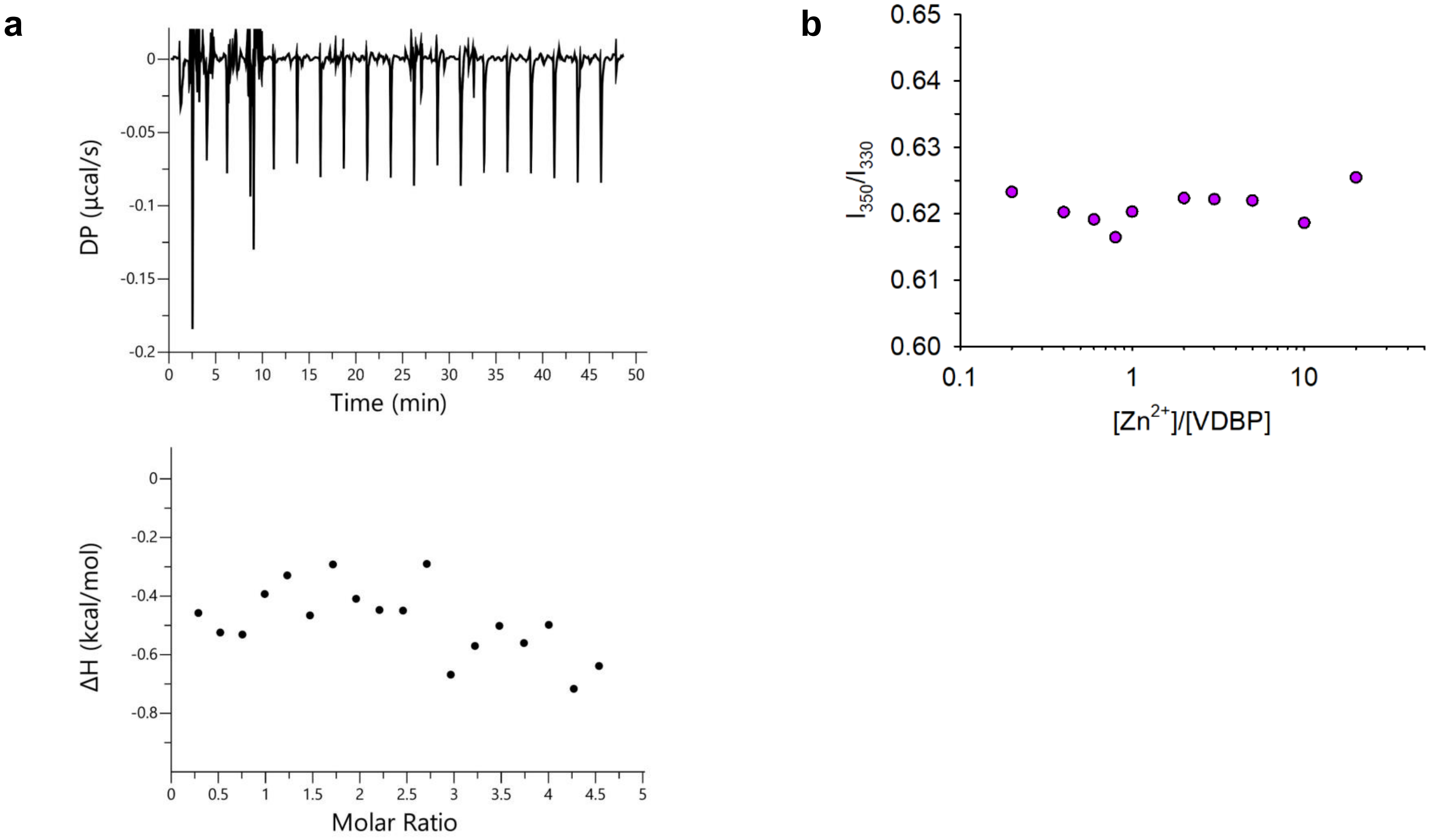


**Supplementary Figure 2**. Zinc complexation with VDBP was examined by ITC (**a**) and nanoDSF (**b**). Zinc titration does not lead to thermodynamic effects and does not affect tryptophan fluorescence of VDBP indicating the absence of zinc binding to the protein.

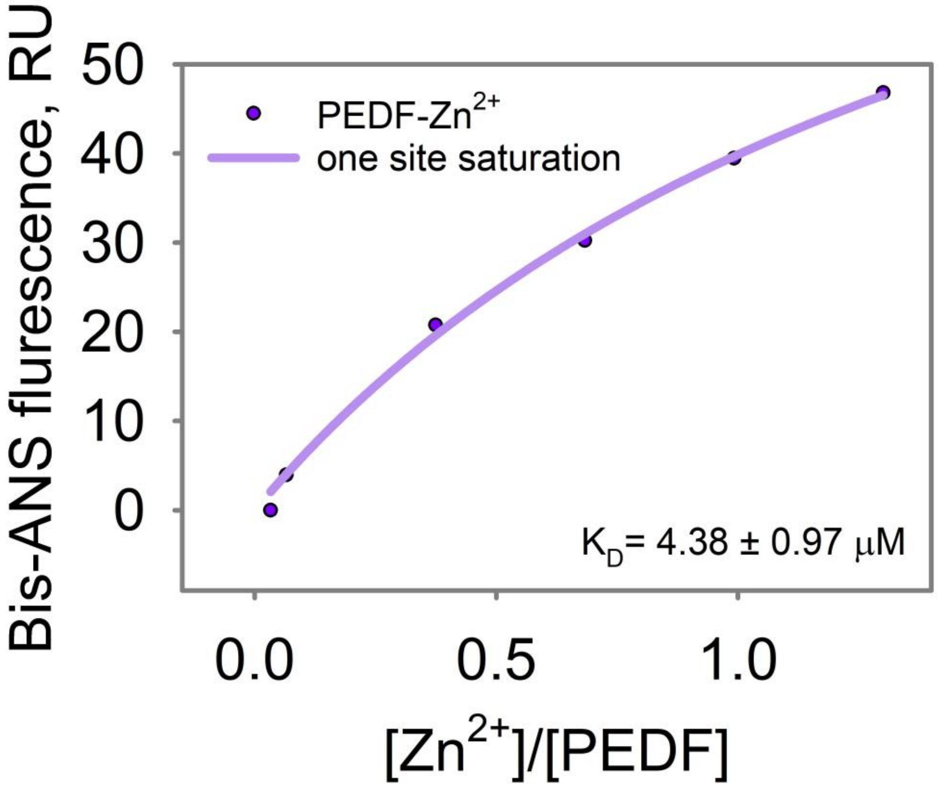


**Supplementary Figure 3**. Formation of a high-affinity 1:1 PEDF-$Zn^{2+}$ complex was monitored by bis-ANS assay. The fluorescence intensity of bis-ANS was plotted against the zinc/PEDF ratio up to a zinc excess of 1.4. The experimental data were fitted to a 2-parameter single-site saturation model. The apparent dissociation constant ($K_D$) was calculated from this model.

**a** **b**

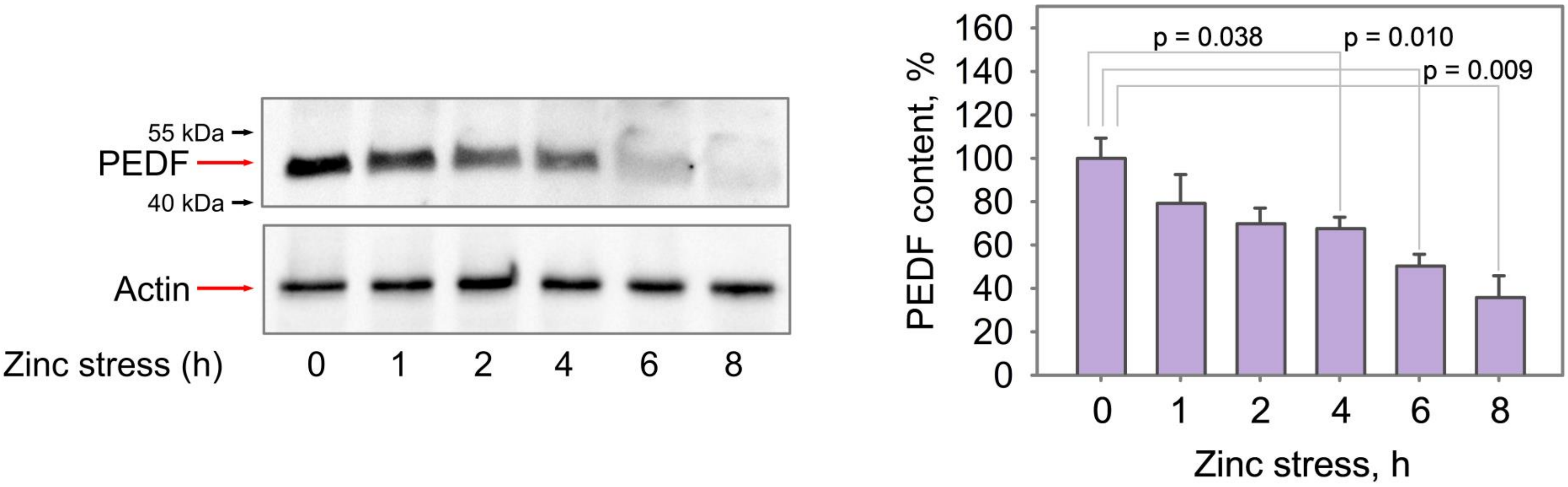


**Supplementary Figure 4**. Effect of zinc stress on PEDF content in non-damaged Y79 cells was analyzed by Western blotting. The amount of the total protein in each track was adjusted using actin as a loading control (**a**). Weight fractions of PEDF estimated from the Western blotting data from at least three independent measurements (**b**). The amount of PEDF in the absence of stress was taken as 100%.

**a**

**b**

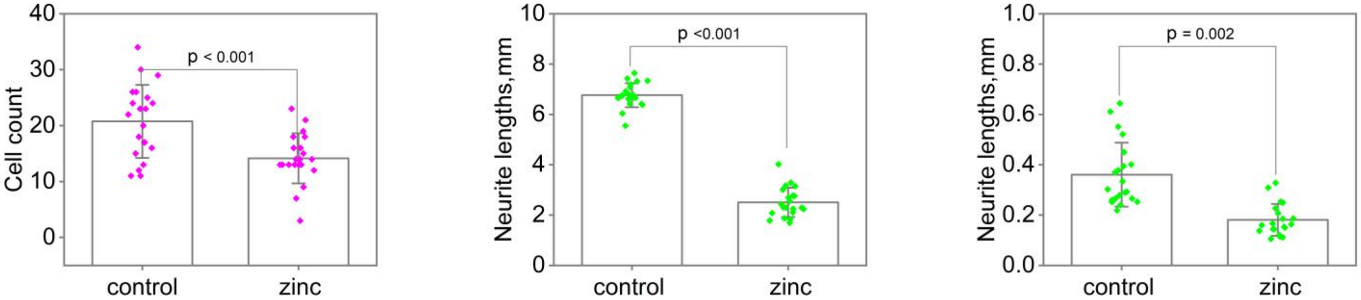


**Supplementary Figure 5**. Effect of zinc (100 μM) on viability and neurite dropout in differentiated SH-SY5Y cells. Cell count, total neurite length and neurite length per cell were determined from confocal microscopy data with nuclei staining by Hoechst 33342 and neurites staining with antibodies against Synapsin I/II/III (scale bar = 25 μm, n=20).

```
tr|G1SCK5|G1SCK5_RABIT      MQALVLLLWTGALLGHGSCQSIASSPEEGSPAPDGTGPPLEEDDPFFKVPVNKLAAAVSN   60
sp|P97298|PEDF_MOUSE        MQALVLLLWTGALLGHGSSQNVPS-SSEGSPVPDSTGEPVEEEDPFFKVPVNKLAAAVSN   59
sp|Q95121|PEDF_BOVIN        MQALVLLLWTGALLGFGRCQNAG--QEAGSLTPESTGAPVEEEDPFFKVPVNKLAAAVSN   58
sp|P36955|PEDF_HUMAN        MQALVLLLCIGALLGHSSCQNPASPPEEGSPDPDSTGALVEEEDPFFKVPVNKLAAAVSN   60
                            ********  *****.. .*.     . **  *:.**  :**:*****************

tr|G1SCK5|G1SCK5_RABIT      FGYDLYRQRSAESPTANVLLSPLSVATALSALSLGAEQRTESLIQRALYYDLISNPDIHG   120
sp|P97298|PEDF_MOUSE        FGYDLYRLRSSASPTGNVLLSPLSVATALSALSLGAEHRTESVIHRALYYDLITNPDIHS   119
sp|Q95121|PEDF_BOVIN        FGYDLYRVRSGESPTANVLLSPLSVATALSALSLGAEQRTESNIHRALYYDLISNPDIHG   118
sp|P36955|PEDF_HUMAN        FGYDLYRVRSSTSPTTNVLLSPLSVATALSALSLGAEQRTESIIHRALYYDLISSPDIHG   120
                            ******* **. *** *********************:**** *:********:.****.

tr|G1SCK5|G1SCK5_RABIT      TYKELLAAVTAPQKNLKSASRIIFERKLRIKSSFVAPLEKAYGTRPRVLSGNPRLDLQEL   180
sp|P97298|PEDF_MOUSE        TYKELLASVTAPEKNLKSASRIVFERKLRVKSSFVAPLEKSYGTRPRILTGNPRVDLQEI   179
sp|Q95121|PEDF_BOVIN        TYKDLLASVTAPQKNLKSASRIIFERKLRIKASFIPPLEKSYGTRPRILTGNSRVDLQEI   178
sp|P36955|PEDF_HUMAN        TYKELLDTVTAPQKNLKSASRIVFEKKLRIKSSFVAPLEKSYGTRPRVLTGNPRLDLQEI   180
                            ***:** :****:*********:**:***:*:**: ****:******:*:** *:****:

tr|G1SCK5|G1SCK5_RABIT      NGWVQAQMKGKVARSAREVPSDISIFLLGVAYFKGQWVTKFDSRKTSLQDFHLDEERTVR   240
sp|P97298|PEDF_MOUSE        NNWVQAQMKGKIARSTREMPSALSILLLGVAYFKGQWVTKFDSRKTTLQDFHLDEDRTVR   239
sp|Q95121|PEDF_BOVIN        NNWVQAQMKGKVARSTREMPSEISIFLLGVAYFKGQWVTKFDSRKTSLEDFYLDEERTVK   238
sp|P36955|PEDF_HUMAN        NNWVQAQMKGKLARSTKEIPDEISILLLGVAHFKGQWVTKFDSRKTSLEDFYLDEERTVR   240
                            *.*********:***::*:*. :**:*****:**************:*:**:***:***:

tr|G1SCK5|G1SCK5_RABIT      VPMMSDPKATLRYGLDSDLNCKIAQLPLTGSMSIIFFLPLRPTQNLTLIEESLTSEFIHD   300
sp|P97298|PEDF_MOUSE        VPMMSDPKAILRYGLDSDLNCKIAQLPLTGSMSIIFFLPLTVTQNLTMIEESLTSEFIHD   299
sp|Q95121|PEDF_BOVIN        VPMMSDPQAVLRYGLDSDLNCKIAQLPLTGSTSIIFFLPQKVTQNLTLIEESLTSEFIHD   298
sp|P36955|PEDF_HUMAN        VPMMSDPKAVLRYGLDSDLSCKIAQLPLTGSMSIIFFLPLKVTQNLTLIEESLTSEFIHD   300
                            *******:* *********.*********** *******   *****:************

tr|G1SCK5|G1SCK5_RABIT      IDRELKTIQAALSIPRLKLSFEGEVTKALQEIKLQPLFESPDFSKITGKPIKLTHVEHRA   360
sp|P97298|PEDF_MOUSE        IDRELKTIQAVLTVPKLKLSFEGELTKSLQDMKLQSLFESPDFSKITGKPVKLTQVEHRA   359
sp|Q95121|PEDF_BOVIN        IDRELKTVQAVLTIPKLKLSYEGELTKSVQELKLQSLFDAPDFSKITGKPIKLTQVEHRV   358
sp|P36955|PEDF_HUMAN        IDRELKTVQAVLTVPKLKLSYEGEVTKSLQEMKLQSLFDSPDFSKITGKPIKLTQVEHRA   360
                            *******:**.*::*:****:***:**::*::*** **::**********:***:****.

tr|G1SCK5|G1SCK5_RABIT      GFEWNEDGAATSPGPEVQPARLAFSLDYHLNQPFIFVLRDSDTGALLFVGKILDPRGT   418
sp|P97298|PEDF_MOUSE        AFEWNEEGAGSSPSPGLQPVRLTFPLDYHLNQPFLFVLRDTDTGALLFIGRILDPSST   417
sp|Q95121|PEDF_BOVIN        GFEWNEDGAGTNSSPGVQPARLTFPLDYHLNQPFIFVLRDTDTGALLFIGKILDPRGT   416
sp|P36955|PEDF_HUMAN        GFEWNEDGAGTTPSPGLQPAHLTFPLDYHLNQPFIFVLRDTDTGALLFIGKILDPRGP   418
                            .*****:**.:. .* :**.:*:* *********:*****:*******:*:**** .
```

**Supplementary Figure 6**. Multiple sequence alignment of mammlian PEDF (SERPINF1) using CLUSTAL O(1.2.4). Amino acid residues corresponding to the zinc coordinators found in the crystal structure of human PEDF (see Figure 4) are marked in green (matching), yellow (similar), or red (different).

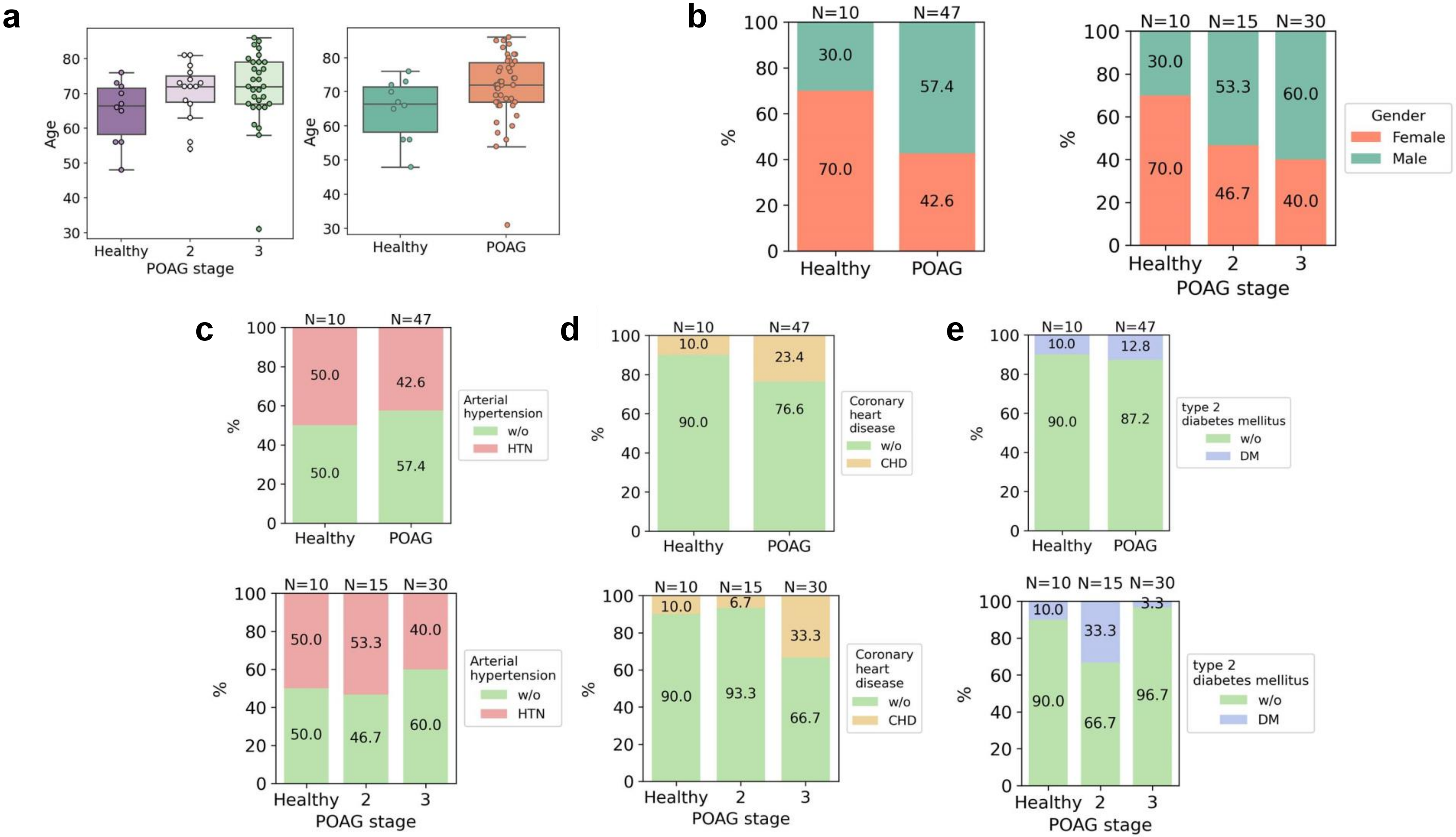


**Supplementary Figure 7**. Comparison of demographic characteristics and comorbidities in groups of the study participants performed using one-way ANOVA in the case of numeric parameters (age) (**a**) or Fisher's exact test in the case of categorical variables (gender (**b)** and comorbidities (**c-e**)). P-values are shown in Table S2.

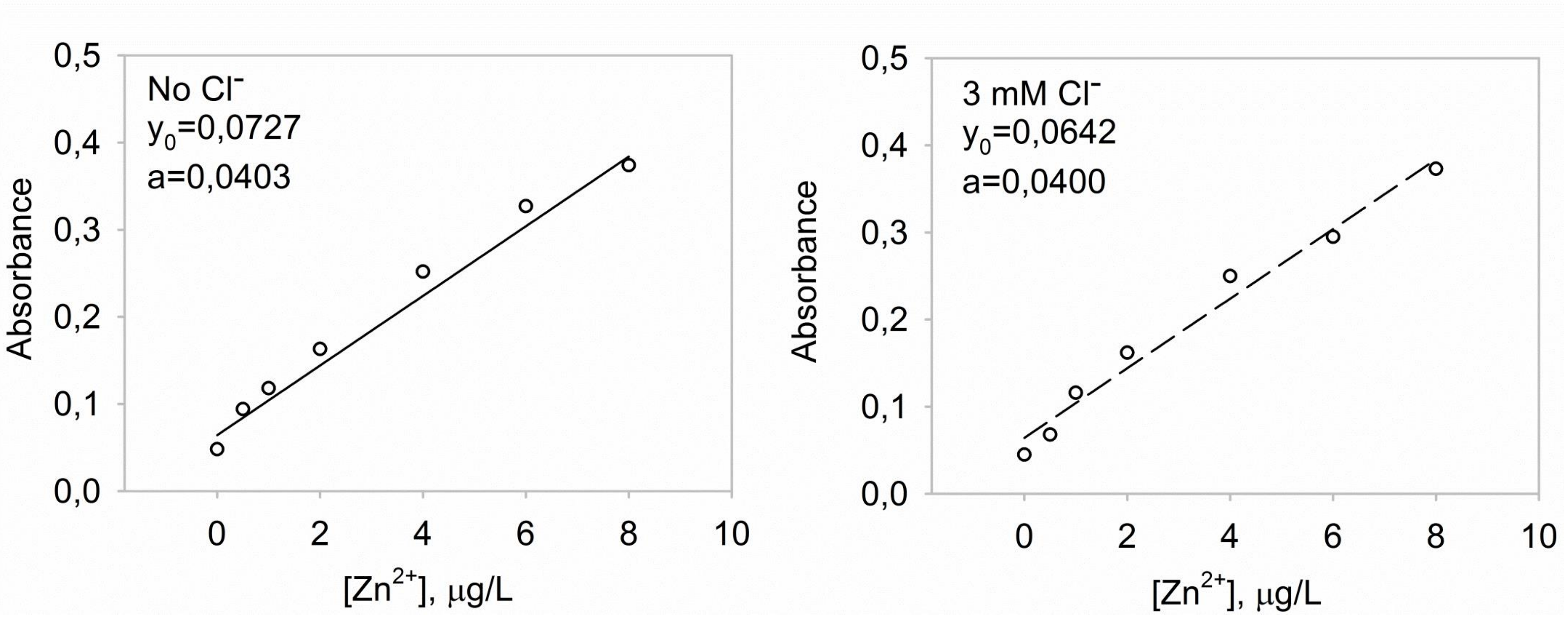


**Supplementary Figure 8**. Calibration plots obtained in the range of 0.5-8 μg/L $Zn^{2+}$ with or without 3 mM $Cl^{-}$. Zinc content was measured using AAS with electrothermal atomization and Zeeman background correction.

**a**

```
        10         20         30         40         50         60         70         80         90        100        110        120
LLSPLSVATA LSALSLGAEQ RTESIIHRAL YYDLISSPDI HGTYKELLDT VTAPQKNLKS MQNPASPPEE GSPDPDSTGA LVEEEDPFFK VPVNKLAAAV SNFGYDLYRV RSSTSPTTNV
       130        140        150        160        170        180        190        200        210        220        230        240
IPDEISILLL GVAHFKGQWV TKFDSRKTSL EDFYLDEERT VRVPMMSDPK AVLRYGLDSD ASRIVFEKKL RIKSSFVAPL EKSYGTRPRV LTGNPRLDLQ EINNWVQAQM KGKLARSTKE
       250        260        270        280        290        300        310        320        330        340        350        360
LSYEGEVTKS LQEMKLQSLF DSPDFSKITG KPIKLTQVEH RAGFEWNEDG AGTTPSPGLQ LSCKIAQLPL TGSMSIIFFL PLKVTQNLTL IEESLTSEFI HDIDRELKTV QAVLTVPKLK
       370        380        390        400
PAHLTFPLDY HLNQPFIFVL RDTDTGALLF IGKILDPRGP
```

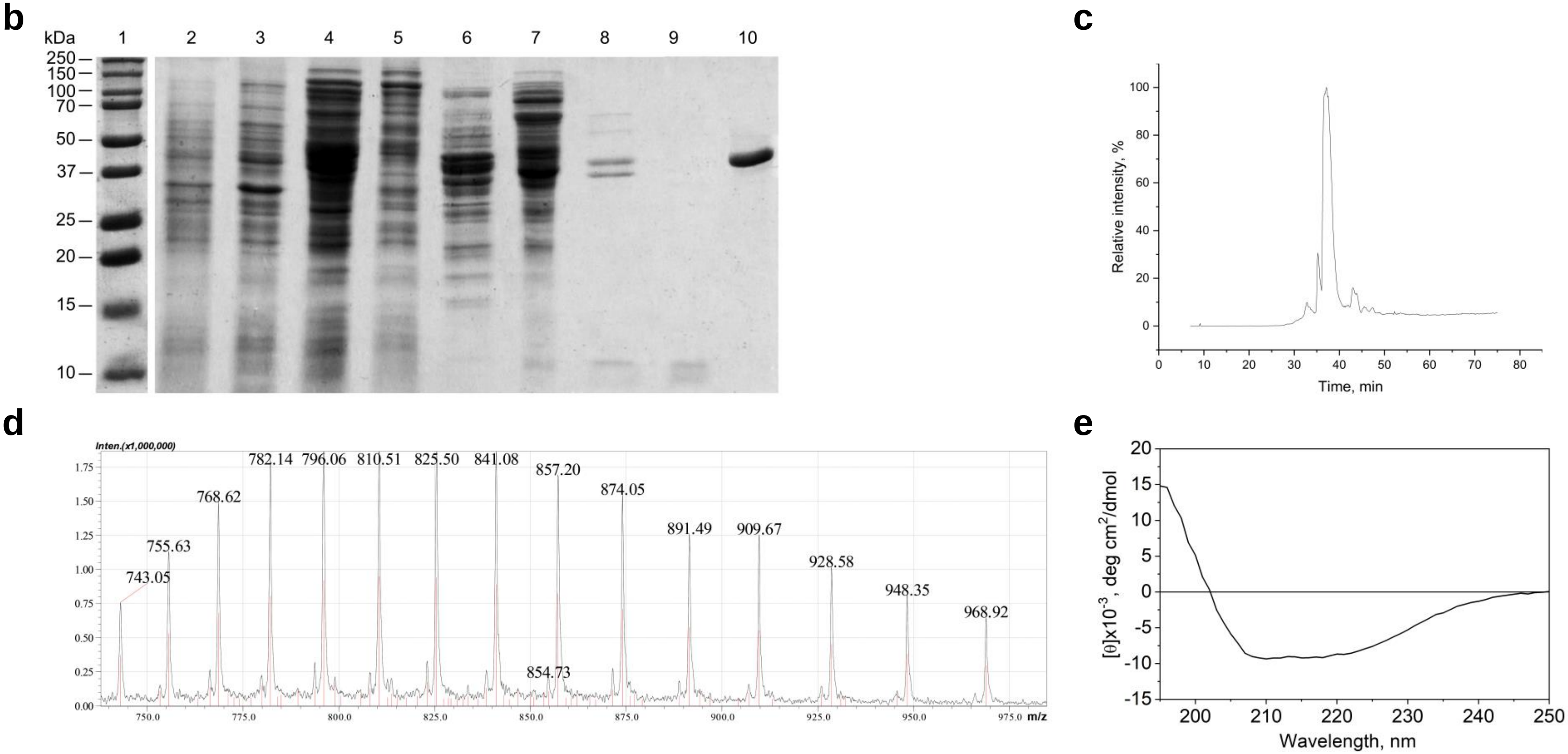


**Supplementary Figure 9**. (**a**) Sequence of recombinant human PEDF obtained in this study. (**b**) SDS-PAGE of fractions obtained during PEDF expression in E.coli BL21(DE3)Star cells and purification from bacterial lysates. Line 1 – protein ladder, line 2 – cells before induction, line 3 – cells after induction, line 4 – soluble proteins isolated from cell debris, line 5 – proteins unbound to DEAE-Sepharose, line 6 – PEDF-containing eluate from DEAE-Sepharose, line 7 – proteins unbound to heparin-Sepharose, line 8 – PEDF-containing eluate from heparin-Sepharose, line 9 – proteins unbound to MonoQ, line 10 – PEDF-containing fraction eluate from MonoQ. HPLC (C18) profile (**b**), mass-spectrum (**d**) and far-UV CD spectrum (**e**) of purified recombinant PEDF.

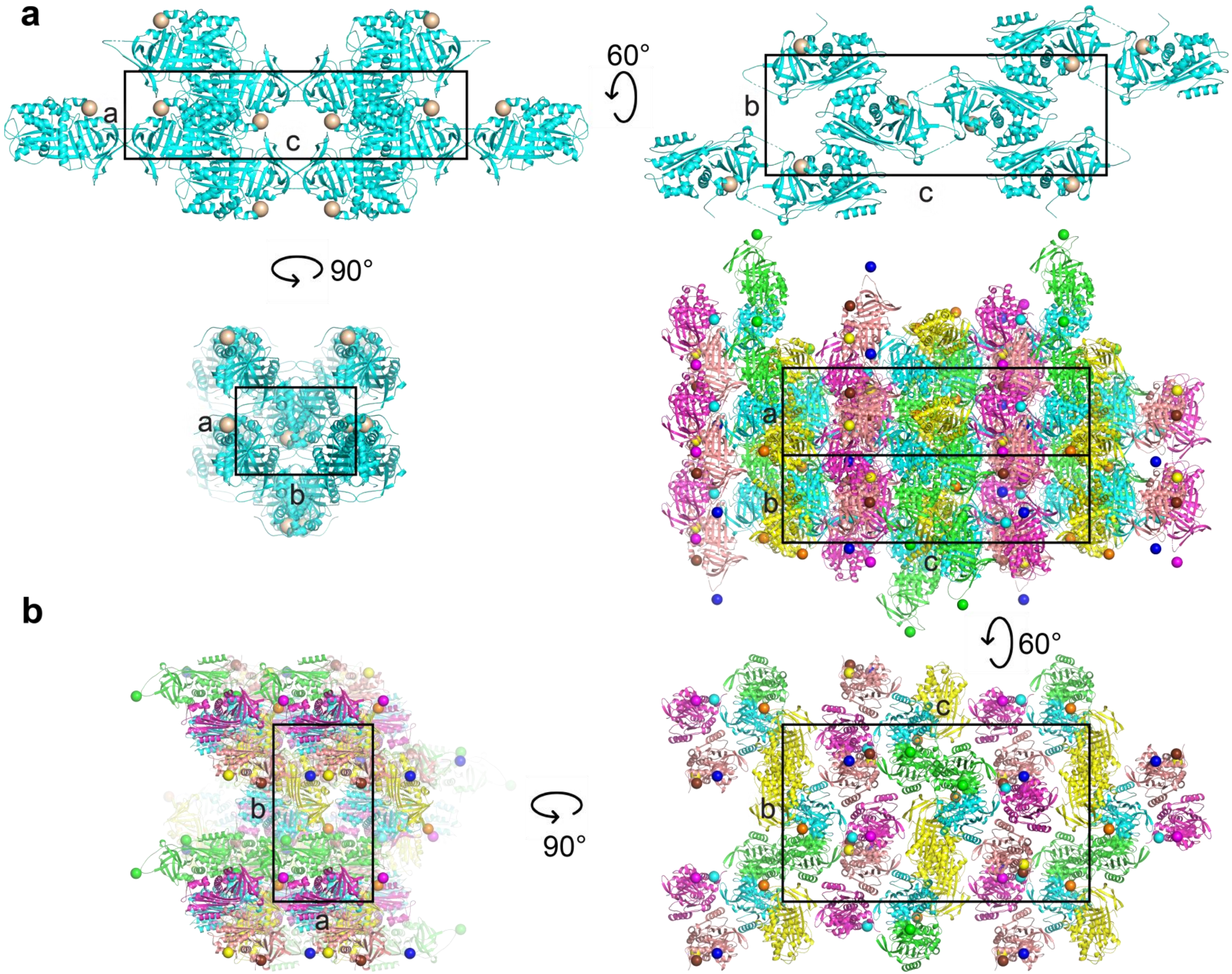


**Supplementary Figure 10.** Crystal packing of PEDF molecules in $P2_12_12_1$ (**a**) and $P22_12_1$ (**b**). Different chains are shown in various colors. The colors of $Zn^+$ ions correspond to Fig. 4.

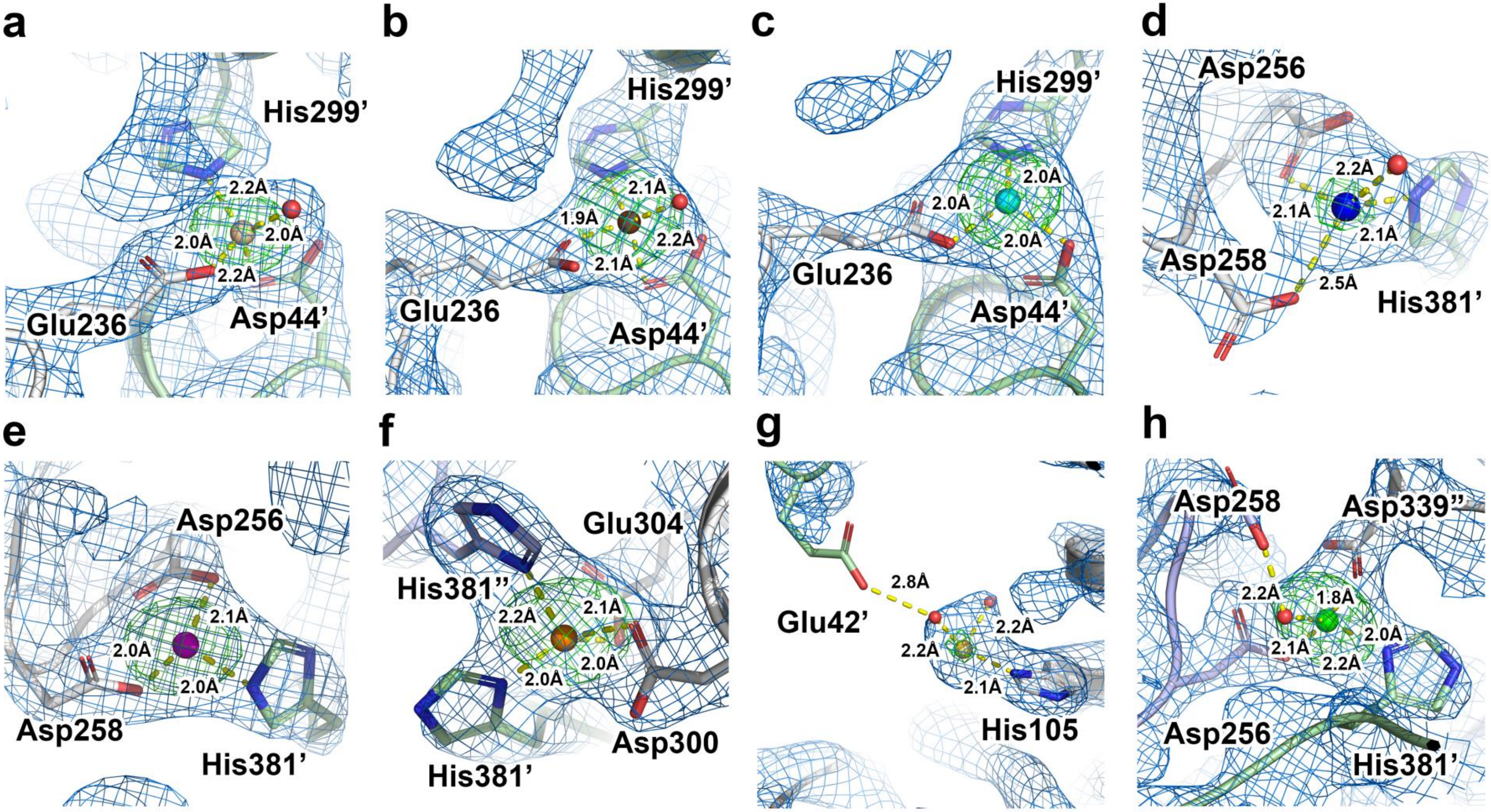


**Supplementary Figure 11**. Electron density maps of PEDF Zn-binding sites. Panels **a-h** correspond to sites 1-8. 2F*o*-F*c* maps and anomalous difference maps are shown in blue and green at 1.3σ and 7σ, respectively. The colors of $Zn^{+}$ ions and protein molecules correspond to Fig. 4.

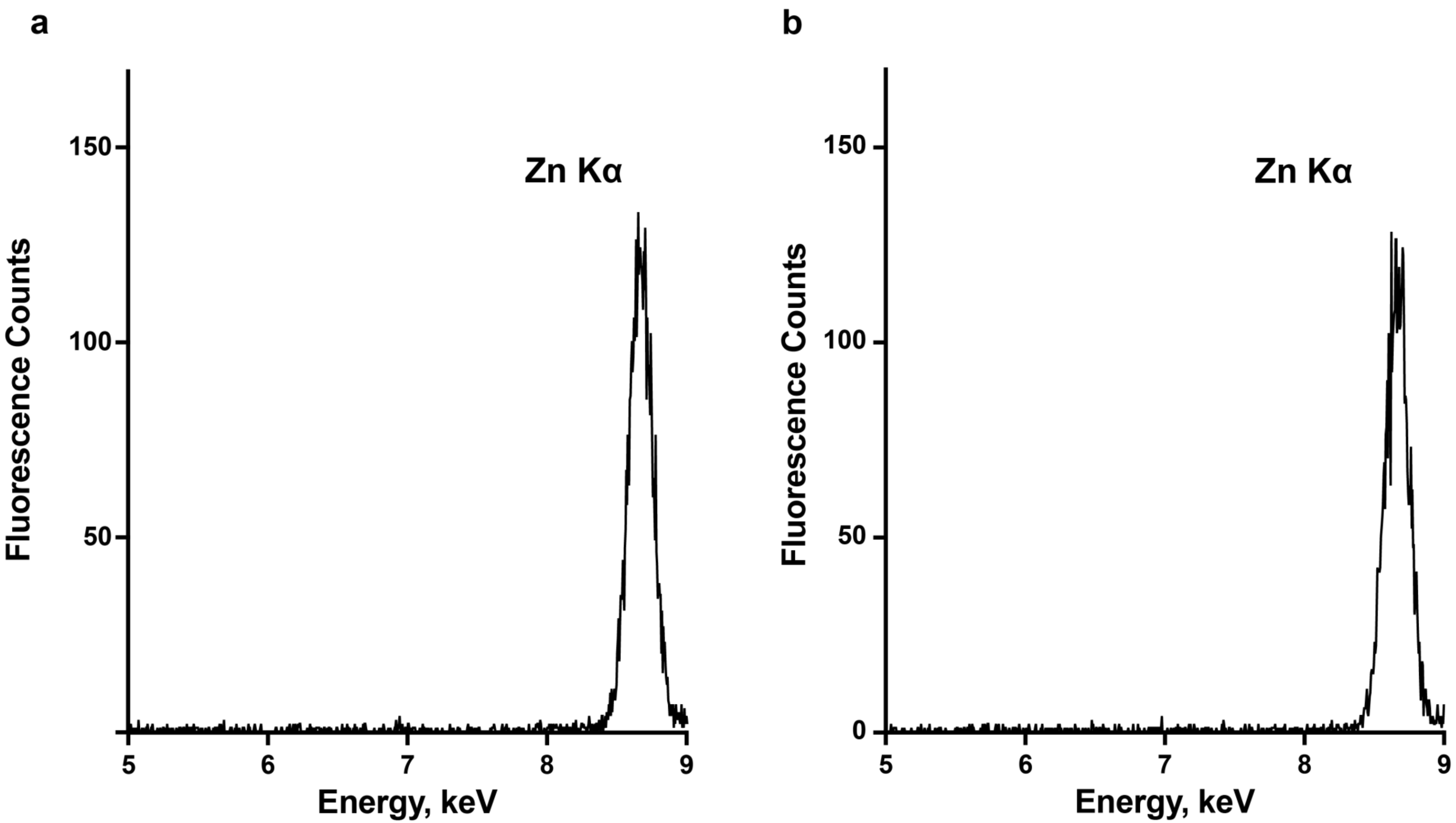


**Supplementary Figure 12**. X-Ray Fluorescence spectrum of PEDF $P22_12_1$ (a) and $P2_12_12_1$ (b) crystals with λ=1.24Å incident beam.

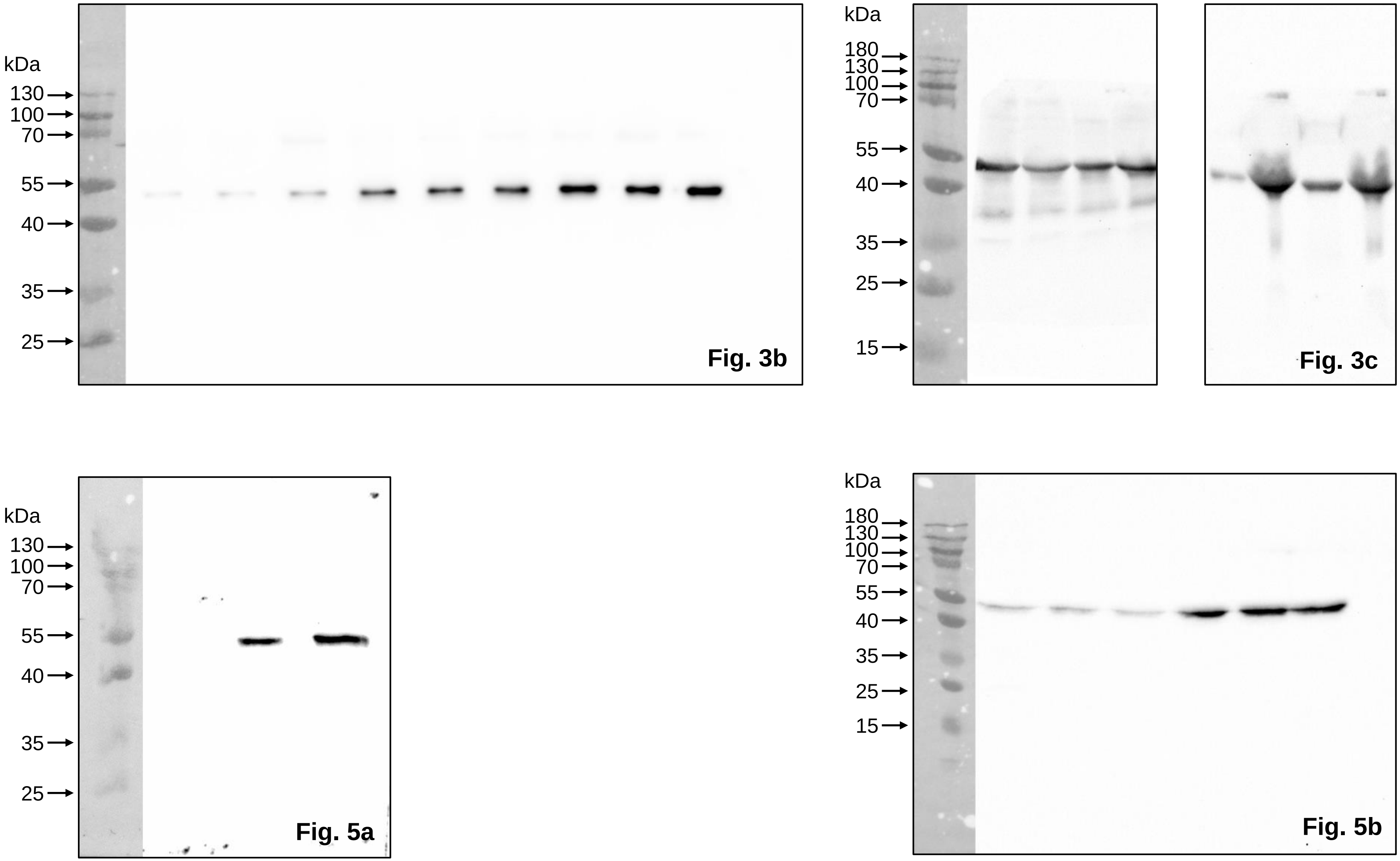


**Supplementary Figure 13**. Full-length Western blot images presented in Fig. 3, b-c and Fig. 5, a-b.

.
