# Supplementary material for: A role of pigment epithelium-derived factor in zinc-mediated mechanism of neurodegeneration in glaucoma
Source: Commun Biol. 2025 Jul 1;8:965. doi: 10.1038/s42003-025-08370-8 (PMC12215840; doi:10.1038/s42003-025-08370-8)
Supplement: Supplementary file 2 — Description of Additional Supplementary Files [file 42003_2025_8370_MOESM2_ESM.pdf]

Description of Additional Supplementary Files

File name: Supplementary Data
Description: Supplementary Data 1-15 presented as a single xlsx-file.

Supplementary Data 1
Description: Characteristics of control and POAG groups

Supplementary Data 2
Description: Comparison of demographic characteristics and comorbidities in patient groups

Supplementary Data 3
Description: Effect of antiglaucomatous therapy on zinc content in AH

Supplementary Data 4
Description: Nomenclature of identified AH metabolites

Supplementary Data 5
Description: AH metabolites characterized by significant ($p < 0.05$ and/or VIP score > 1) changes in stage 2 POAG

Supplementary Data 6
Description: AH metabolites characterized by significant ($p < 0.05$ and/or VIP score > 1) changes in stage 3 POAG

Supplementary Data 7
Description: AH metabolites characterized by significant ($p < 0.05$ and/or VIP score > 1) changes in total POAG

Supplementary Data 8
Description: Summary of AH metabolites characterized by significant changes in POAG

Supplementary Data 9
Description: Effect of antiglaucomatous therapy on metabolites content in AH

Supplementary Data 10
Description: AH proteins assigned to the constitutive AH proteome and presented in the PDB database containing potential Zn^{2+}-binding sites as predicted by the ZincBindPredict program

Supplementary Data 11
Description: AH proteins assigned to the constitutive AH proteome and presented in the PDB and AlphaFold databases containing potential Zn^{2+}-binding sites as predicted by the ZincBindPredict program

Supplementary Data 12
Description: Optimized AAS measurement program

Supplementary Data 13
Description: Summary of the data processing statistics. The values in parentheses refer to the highest-resolution shell. Friedel's pairs are unmerged.

Supplementary Data 14
Description: Crystallographic data and refinement statistics for the final models.

Supplementary Data 15
Description: The source data underlying the plots in the main figures.
