# Supplementary material for: A role of pigment epithelium-derived factor in zinc-mediated mechanism of neurodegeneration in glaucoma
Source: Commun Biol. 2025 Jul 1;8:965. doi: 10.1038/s42003-025-08370-8 (PMC12215840; doi:10.1038/s42003-025-08370-8)
Supplement: Supplementary file 4 — Reporting Summary [file 42003_2025_8370_MOESM4_ESM.pdf]

nature portfolio

Corresponding author(s): Evgeni Yu. Zernii

Last updated by author(s): May 25, 2025

Reporting Summary

Nature Portfolio wishes to improve the reproducibility of the work that we publish. This form provides structure for consistency and transparency in reporting. For further information on Nature Portfolio policies, see our Editorial Policies and the Editorial Policy Checklist.

Statistics

For all statistical analyses, confirm that the following items are present in the figure legend, table legend, main text, or Methods section.

n/a	Confirmed	
☐	☒	The exact sample size (*n*) for each experimental group/condition, given as a discrete number and unit of measurement
☐	☒	A statement on whether measurements were taken from distinct samples or whether the same sample was measured repeatedly
☐	☒	The statistical test(s) used AND whether they are one- or two-sided<br>*Only common tests should be described solely by name; describe more complex techniques in the Methods section.*
☐	☒	A description of all covariates tested
☐	☒	A description of any assumptions or corrections, such as tests of normality and adjustment for multiple comparisons
☐	☒	A full description of the statistical parameters including central tendency (e.g. means) or other basic estimates (e.g. regression coefficient) AND variation (e.g. standard deviation) or associated estimates of uncertainty (e.g. confidence intervals)
☐	☒	For null hypothesis testing, the test statistic (e.g. *F*, *t*, *r*) with confidence intervals, effect sizes, degrees of freedom and *P* value noted<br>*Give P values as exact values whenever suitable.*
☒	☐	For Bayesian analysis, information on the choice of priors and Markov chain Monte Carlo settings
☒	☐	For hierarchical and complex designs, identification of the appropriate level for tests and full reporting of outcomes
☒	☐	Estimates of effect sizes (e.g. Cohen's *d*, Pearson's *r*), indicating how they were calculated

Our web collection on statistics for biologists contains articles on many of the points above.

Software and code

Policy information about availability of computer code

Data collection	AH metabolites were idetified based on GC-MS data using the Shimadzu Smart metabolite database (#225-28366, Shimadzu, Japan).<br>Zinc-binding sites in protein structures were predicted using the machine learning-based program ZincBindPredict (https://zincbind.net).<br>X-ray data were processed using XDS software (MPI for Medical Research, Heidelberg, Germany); protein structure was solved using SIMBAD automated pipeline (University of Liverpool, Liverpool, UK); model rearrangement and refinement were performed using ModelCraft (CCP4 Cloud, University of York, York, UK) and phenix.refine (PHENIX, University of California Berkeley, Berkeley, CA, USA), respectively; electron density map analysis and manual model building were performed in COOT (MRC Laboratory of Molecular Biology, Cambridge, UK).
Data analysis	Metabolite data were analyzed using MetaboAnalyst 5.0 software (www.metaboanalyst.ca).<br>Histological preparations were analyzed using Zeiss Zen 2 lite blue edition software (Carl Zeiss, Germany), microphotographs were processed using AxioVision v.3.0 software (Carl Zeiss, Germany), morphometric analysis was performed using Aperio ImageScope software (Leica, Germany).<br>Thermal denaturation curves of proteins were analyzed using MicroCal PEAQ-DSC program (Malvern Panalytical, UK).<br>Protein fluorescence emission spectra were analyzed using LogNormal program (IBR RAS, Pushchino, Russia).<br>NanoDSF data were analyzed using PR.ThermControl program (NanoTemper Technologies, Munich, Germany).<br>DLS data were analyzed using ZS Explorer software version 3.2.1 (Malvern Panalytical, UK).<br>Flow cytometry data were analyzed using FlowJo v. 10.0.7 software (FlowJo, Ashland, OR, USA).<br>Confocal microscope images were analyzed using NIS-Elements (Nikon Corporation, Japan) and ImageJ 121 software.<br>Western blot data were analyzed using GelAnalyzer.2010a software (http://www.gelanalyzer.com/).<br>SigmaPlot 11 program (Systat Software, Germany) was used to construct graphs and histograms and to assess statistical significance.

For manuscripts utilizing custom algorithms or software that are central to the research but not yet described in published literature, software must be made available to editors and reviewers. We strongly encourage code deposition in a community repository (e.g. GitHub). See the Nature Portfolio guidelines for submitting code & software for further information.

Data

Policy information about availability of data

All manuscripts must include a data availability statement. This statement should provide the following information, where applicable:

- Accession codes, unique identifiers, or web links for publicly available datasets
- A description of any restrictions on data availability
- For clinical datasets or third party data, please ensure that the statement adheres to our policy

The mass spectral data files of AH metabolites/zinc chelators associated with POAG identified by GC-MS-based metabolomic analysis are deposited at the Center for Computational Mass Spectrometry (MassIVE MSV000093951; https://doi.org/doi:10.25345/C5M32NM8P). PEDF structures are deposited into the Protein Data Bank under accession codes 9J3Q (P22121 space group) and 9J3P (P212121 space group). The other datasets used and/or analyzed during the current study are available from the corresponding author on reasonable request.

Research involving human participants, their data, or biological material

Policy information about studies with human participants or human data. See also policy information about sex, gender (identity/presentation), and sexual orientation and race, ethnicity and racism.

Reporting on sex and gender	Mean gender ratio (Fisher test p-value 0.2633) of the study participants showed no significant differences between control group and groups with different glaucoma stages nor between control group and total POAG group.
Reporting on race, ethnicity, or other socially relevant groupings	No socially constructed or socially relevant categorization was used. Study participants were termed 'patients' or 'participants'.
Population characteristics	Mean age ratio (one-way ANOVA p = 0.168) of the study participants showed no significant differences between control group and groups with different glaucoma stages nor between control group and total POAG group. The covariate-relevant population characteristics of the participants included antiglaucomatous treatments such as the use of prostaglandin analogs, beta-adrenoblockers, carboanhydrase inhibitors and alpha-2-adrenomimetics.
Recruitment	The study involved patients who underwent surgical treatment at the Helmholtz National Medical Research Center for Eye Diseases. The control group included patients without visual pathology, except for cataract. The experimental groups included patients of similar age with clinically established diagnosis of POAG on the basis of complex examination including tonometry, visometry, biomicroscopy, ophthalmoscopy, perimetry and optical coherence tomography (with estimation of the true thickness of the neuroretinal rim. Additional examination included retinal tomography with determination of the area and volume of the neuroretinal rim, the average thickness of the retinal nerve fiber layer at the disc margin, and the cap-to-disc ratio (C/D). As a result, patients were categorized as stages 2-4 of POAG. Stage 2 (moderate POAG) included individuals with changes in the paracentral visual field (narrowing of more than 10° in the nasal hemifield) and moderate excavation in at least one sector reaching the fundus edge. The mean C/D in these patients was ~0.6-0.7. Stage 3 (advanced glaucoma) included patients with a concentrically narrowed visual field (less than 15° from fixation in at least one segment) and subtotal marginal excavation of the optic nerve. The mean C/D value in patients with this stage was ~0.85. Patients with stage 4 (terminal glaucoma) were characterized by complete excavation of the optic nerve and significant vision loss. Most patients received hypotensive therapy including the use of prostaglandin analogs, beta-adrenoblockers, carboanhydrase inhibitors and/or alpha-2-adrenomimetics for at least 1 year before surgery with IOP control once every 2 months.
Ethics oversight	The studies involving human participants were conducted in accordance with the Declaration of Helsinki and the ARVO statement and were approved by the local ethical committee of the Helmholtz National Medical Research Center for Eye Diseases (No. 58, 17.03.2022). All participants signed written informed consent.

Note that full information on the approval of the study protocol must also be provided in the manuscript.

Field-specific reporting

Please select the one below that is the best fit for your research. If you are not sure, read the appropriate sections before making your selection.

☒ Life sciences ☐ Behavioural & social sciences ☐ Ecological, evolutionary & environmental sciences

For a reference copy of the document with all sections, see nature.com/documents/nr-reporting-summary-flat.pdf

Life sciences study design

All studies must disclose on these points even when the disclosure is negative.

Sample size	In human studies, the total number and distribution of participants were determined by the overall capability and schedule of surgical interventions at the Helmholtz National Medical Research Centre for Eye Diseases and the time available for patient recruitment. In animal studies, sample sizes were determined by the recommendations of the ethical committee. In cases when experiments did not involve euthanasia (AH collection) the size was 8-10 animals in a group. In experiments involving euthanasia (histological analysis of the posterior sector of the eye, obtaining retinal samples), the number of animals in the control (healthy) groups was reduced twofold.

Data exclusions	No data were exculded from the analysis.
Replication	Reproducibility of experiments involving animals was ensured by the sample size (8-10, see above). All in vitro experiments were performed in at least 3 replicates (usually 6-20 replicates).
Randomization	Human study participants were divided into groups according to the established clinical diagnosis (see above). All animals involved in the study were of the same breed, gender (males), age and weight and were divided into experimental and control groups randomly.
Blinding	Experiments on biochemical and histological analyses of human and animal samples were blinded. In the case of in vitro studies, such a requirement was not feasible or unnecessary due to the inability of the researcher to influence the results.

Reporting for specific materials, systems and methods

We require information from authors about some types of materials, experimental systems and methods used in many studies. Here, indicate whether each material, system or method listed is relevant to your study. If you are not sure if a list item applies to your research, read the appropriate section before selecting a response.

Materials & experimental systems

n/a	Involved in the study	
☐	☒	Antibodies
☐	☒	Eukaryotic cell lines
☒	☐	Palaeontology and archaeology
☐	☒	Animals and other organisms
☒	☐	Clinical data
☒	☐	Dual use research of concern
☒	☐	Plants

Methods

n/a	Involved in the study	
☒	☐	ChIP-seq
☐	☒	Flow cytometry
☒	☐	MRI-based neuroimaging

Antibodies

Antibodies used	Polyclonal anti-PEDF antibodies were from Cloud-Clone corp., China (#PAB972Hu01).<br>Alexa Fluor® 488 anti-Synapsin I/II/III Antibodies (clone A17080A) were form Fisher Scientific, USA (Catalog No. 50-207-4722; Supplier: Biolegend # 853712).<br>Antibodies against β-III tubulin conjugated to Alexa Fluor® 647 were from Biolegend, USA (Catalog No. 657405).
Validation	Anti-PEDF antibodies have been validated in studies published in Molecular Therapy (Cell press, PMID: 20647999), Investigative ophthalmology & visual science (PMIDs: 27820950, 30025136) and other journals (summary on the manufacturer's website https://www.cloud-clone.com/products/PAB972Hu01.html).<br>Antibodies against synapsin I/II/III (clone A17080A) have been validated in studies published in Cancer Cell (Cell press, PMID: 31631026), FASEB Bioadvances (PMID: 35664831), Metallomics (PMID: 38936837) and other journals.<br>Antibodies against β-III tubulin conjugated to Alexa Fluor® 647 have been validated in studies published in Nature Neuroscience (PMID: 34059832), FeLife (PMID: 29993362), Molecular cell (PMID: 32707033) and other journals.

Eukaryotic cell lines

Policy information about cell lines and Sex and Gender in Research

Cell line source(s)	Human retinoblastoma Y79 cells were from DSMZ-German Collection of Microorganisms and Cell Cultures GmbH, Germany (#ACC-246).<br>Human retinal pigment epithelium ARPE-19 cells were form Cell Culture Collection of the Koltzov Institute of Developmental Biology of Russian Academy of Sciences, Russia.<br>Human neuroblastoma SH-SY5Y cells were from American Type Culture Collection, USA (#CRL-2266).
Authentication	None of the cell lines used were authenticated.
Mycoplasma contamination	All cell lines were tested negative for mycoplasma contamination.
Commonly misidentified lines (See ICLAC register)	n/a

Animals and other research organisms

Policy information about studies involving animals; ARRIVE guidelines recommended for reporting animal research, and Sex and Gender in Research

Laboratory animals	Rabbits, New Zealand white, 4 months old.

Wild animals | n/a

Reporting on sex | The study involved only male rabbits.

Field-collected samples | n/a

Ethics oversight | Animal studies were performed according to the 8th edition “Guide for the Care and Use of Laboratory Animals” of the National Research Council and “Statement for the Use of Animals in Ophthalmic and Visual Research” of The Association for Research in Vision and Ophthalmology (ARVO). The protocol was approved by Bioethics Commission of Belozersky Institute of Physico-chemical Biology and Faculty of Bioengineering and Bioinformatics of Lomonosov Moscow State University (No. 005-5/9/2023, 30.06.2023).

Note that full information on the approval of the study protocol must also be provided in the manuscript.

Plants

Seed stocks | n/a

Novel plant genotypes | n/a

Authentication | n/a

Flow Cytometry

Plots

Confirm that:

☒ The axis labels state the marker and fluorochrome used (e.g. CD4-FITC).

☒ The axis scales are clearly visible. Include numbers along axes only for bottom left plot of group (a 'group' is an analysis of identical markers).

☒ All plots are contour plots with outliers or pseudocolor plots.

☒ A numerical value for number of cells or percentage (with statistics) is provided.

Methodology

Sample preparation | Zn-induced apoptosis in Y79 cells was measured by flow cytometry. Prior to analysis, Y79 cells in a 6-well plate (7.5×10^5 cells per well) were cultured in RPMI-1640 medium supplemented with 20% FBS for 72 h. Cells were harvested by centrifugation (200 g, 5 min, RT), washed with DPBS, resuspended in an initial volume of serum-free RPMI-1640 medium, and cultured for 2 h for serum deprivation. Sterile aqueous ZnSO4 solution was then added and cells were cultured until the indicated time points. Cells were then harvested by centrifugation (200 g, 5 min, RT), washed with ice-cold DPBS, the pellet was resuspended in DPBS and stained with PE annexin V according to the manufacturer's instructions (PE Annexin V Apoptosis Detection kit, BD Biosciences, USA). The stained cells were subjected to flow cytometric analysis always within 1 h after staining.

Instrument | MACSQuant Analyzer 10 flow cytometer (Miltenyi Biotec, USA).

Software | FlowJo v. 10.0.7 (FlowJo, Ashland, OR, USA).

Cell population abundance | n/a (cells analyzed represent a homogeneous population of an established Y79 cell line)

Gating strategy | Y79 cells population was gated on FSC-A/SSC-A dot plot, excluding the cell debris in the lower left corner, and further plotted onto SSC-A/PE-A dot plot. Cells harvested prior addition of ZnSO4 (time point 0 h) were used as a Annexin V-negative population to set up (on the right side) an Annexin V-positive gate in order to monitor an increase in apoptotic (PE Annexin V-positive) cells harvested at a later time points in comparison to untreated cells.

☐ Tick this box to confirm that a figure exemplifying the gating strategy is provided in the Supplementary Information.
